# *ESR1* Is Co-Expressed with Closely Adjacent Uncharacterised Genes Spanning a Breast Cancer Susceptibility Locus at 6q25.1

**DOI:** 10.1371/journal.pgen.1001382

**Published:** 2011-04-28

**Authors:** Anita K. Dunbier, Helen Anderson, Zara Ghazoui, Elena Lopez-Knowles, Sunil Pancholi, Ricardo Ribas, Suzanne Drury, Kally Sidhu, Alexandra Leary, Lesley-Ann Martin, Mitch Dowsett

**Affiliations:** 1 Royal Marsden Hospital, London, United Kingdom; 2 Breakthrough Breast Cancer Research Centre, Institute of Cancer Research, London, United Kingdom; University of Washington, United States of America

## Abstract

Approximately 80% of human breast carcinomas present as oestrogen receptor α-positive (ER+ve) disease, and ER status is a critical factor in treatment decision-making. Recently, single nucleotide polymorphisms (SNPs) in the region immediately upstream of the ER gene (*ESR1*) on 6q25.1 have been associated with breast cancer risk. Our investigation of factors associated with the level of expression of *ESR1* in ER+ve tumours has revealed unexpected associations between genes in this region and *ESR1* expression that are important to consider in studies of the genetic causes of breast cancer risk. RNA from tumour biopsies taken from 104 postmenopausal women before and after 2 weeks treatment with an aromatase (oestrogen synthase) inhibitor was analyzed on Illumina 48K microarrays. Multiple-testing corrected Spearman correlation revealed that three previously uncharacterized open reading frames (ORFs) located immediately upstream of *ESR1*, *C6ORF96*, *C6ORF97*, and *C6ORF211* were highly correlated with *ESR1* (Rs = 0.67, 0.64, and 0.55 respectively, FDR<1×10^−7^). Publicly available datasets confirmed this relationship in other groups of ER+ve tumours. DNA copy number changes did not account for the correlations. The correlations were maintained in cultured cells. An ERα antagonist did not affect the ORFs' expression or their correlation with *ESR1*, suggesting their transcriptional co-activation is not directly mediated by ERα. siRNA inhibition of *C6ORF211* suppressed proliferation in MCF7 cells, and *C6ORF211* positively correlated with a proliferation metagene in tumours. In contrast, *C6ORF97* expression correlated negatively with the metagene and predicted for improved disease-free survival in a tamoxifen-treated published dataset, independently of *ESR1*. Our observations suggest that some of the biological effects previously attributed to ER could be mediated and/or modified by these co-expressed genes. The co-expression and function of these genes may be important influences on the recently identified relationship between SNPs in this region and breast cancer risk.

## Author Summary

Recent genome-wide analysis has revealed that the way in which genes are arranged on chromosomes and the conformation of these chromosomes are crucial for the regulation of gene expression. Reflecting this arrangement, clusters of genes which are regulated together have been discovered. We have identified a previously unreported transcriptional activity hub spanning *ESR1*, the gene encoding the important breast cancer biomarker oestrogen receptor. Genetic variants immediately upstream of *ESR1* have recently been linked to breast cancer risk. We found that three open reading frames within this region are tightly co-expressed with *ESR1*. We investigated the function of these genes and discovered that one of these co-expressed genes, *C6ORF211*, affects proliferation in cultured cells and is correlated with proliferation in breast tumours. Another of the genes, *C6ORF97*, is negatively correlated with proliferation in breast tumours and predicts for outcome on the anti-oestrogen drug tamoxifen. These findings suggest that the genes could contribute to the phenotype associated with oestrogen-receptor positivity. In addition, they may be involved in the mechanism by which genetic variation in this region of the genome contributes to breast cancer susceptibility.

## Introduction

Breast cancer is the most common malignancy in women, accounting for more than 400,000 deaths per year worldwide [1]. Approximately 80% of human breast carcinomas present as oestrogen receptor α-positive (ER+ve) disease and ER status is arguably the most clinically important biological factor in all oncology [2]. The major molecular features of breast cancer segregate differentially between ER+ve and ER−ve tumours [3], [4]. Tumours which express ERα have been termed luminal type [3], [5] and are associated with response to antioestrogen therapy and improved survival, although the mechanisms by which oestrogen receptor dictates tumour status are poorly understood.

Recent genome wide studies have identified SNPs around *C6ORF97*, an open reading frame (ORF) immediately upstream of the gene encoding ER (*ESR1*) to be associated with increased risk of breast cancer. Zheng *et al.* found that heterozygosity at rs2046210, a SNP in the region between *C6ORF97* and *ESR1*, increased breast cancer risk by an odds ratio of 1.59 in a Chinese population and that this risk was also present in a European population, albeit to a weaker extent [6]. Easton and colleagues confirmed the risk associated with this SNP and reported an at least partly independent risk associated with a second adjacent SNP (rs3757318) in intron 7 of *C6ORF97* [7]. Using ancestry-shift refinement mapping, Stacey *et al.* closed in on the identification of the pathogenic variant and found that the risk allele of a novel SNP in this region (rs77275268), disrupts a partially methylated CpG sequence within a known CTCF binding site [8]. More recently, two further studies have confirmed an association with the region [9], [10]. Our studies have revealed unexpected relationships in the expression patterns in breast carcinomas between *ESR1*, *C6ORF97* and the two genes immediately upstream (*C6ORF211* and *C6ORF96* [*RMND1*]).

Oestrogenic ligands, predominantly oestradiol, are the key mitogens for ER+ve breast cancer. In recent years, high throughput genomic technologies have revealed significant numbers of genes that are expressed in response to oestradiol stimulation *in vitro* [11]–[13] and downregulated in response to oestrogen deprivation in tumours [14]–[16]. Similarly, the transcriptional targets of ERα have been characterised in detail using genome wide chromatin interaction mapping in MCF7 cells [17], [18]. Key oestrogen responsive genes such as *TFF1* and *GREB1* have been shown to be highly responsive to oestradiol stimulation in cell culture models through the binding of ERα to their promoters [19], [20]. Additional genes have been found in hierarchical clustering analyses of ER+ve and ER−ve tumours as part of the so-called “luminal epithelial” gene set characterized by the expression of genes typically expressed in the cells that line the ducts of normal mammary glands including *GATA3* and *FOXA1* [12]. However, the correlates of *ESR1* within an exclusively ER+ve group and the inherent heterogeneity within an exclusively ER+ subgroup remain poorly defined.

Modern, non-steroidal aromatase inhibitors (AIs) are widely used, effective treatments for ER+ve breast cancer [21], [22] and are also excellent pharmacological probes for oestrogen-dependent processes *in vivo* because of their specificity and highly effective suppression of oestrogen synthesis. In this study, we found that the expression of genes in the region immediately upstream of *ESR1* associate strongly with *ESR1* expression in ER+ve primary breast cancers before and after AI treatment and uncover evidence that these associations might impact upon the biological and clinical importance of ERα.

## Results

### *ESR1* expression is correlated with three open reading frames on chromosome 6 in tumours

**Figure 1:**
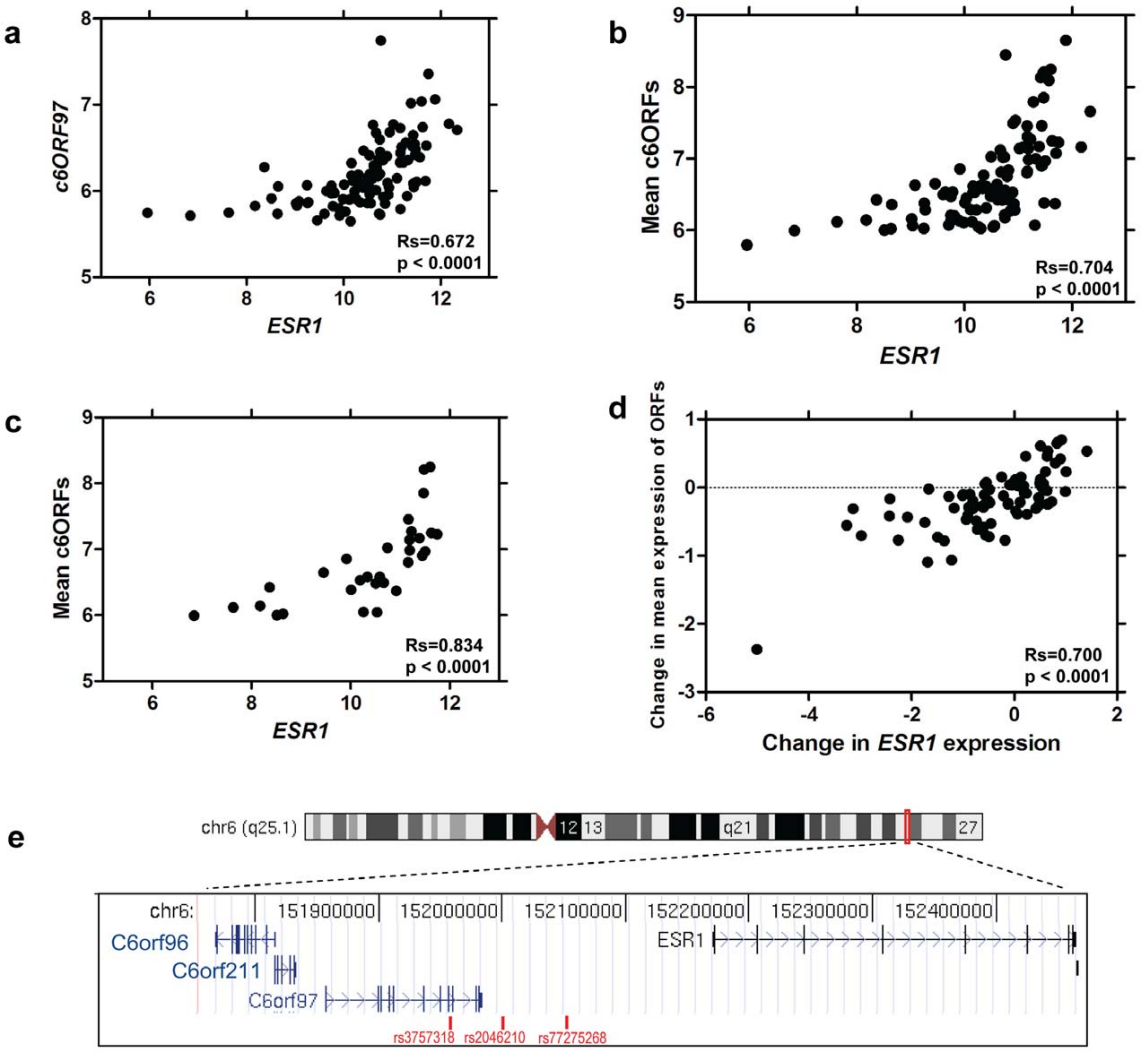
Correlation of *ESR1* expression and oestrogen-responsive gene expression. a. Scatterplot of relationship between expression of *ESR1* and *C6ORF97* in baseline biopsies. b. Correlation between expression of *ESR1* and the mean of *C6ORF96*, *C6ORF97* and *C6ORF211* in baseline biopsies. c. Correlation between *ESR1* and the mean of *C6ORF96*, *C6ORF97* and *C6ORF211* with samples with measured copy number variations shown omitted. d. Scatterplot of relationship between change in *ESR1* and the mean change in *C6ORF96*, *C6ORF97* and *C6ORF211* e. Location of open reading frames, *ESR1* and breast cancer associated SNPs on chromosome 6q25.1.

To investigate correlates of *ESR1*, expression profiles were derived from pairs of 14-guage core cut biopsies before and after 2 weeks' treatment with 1 mg/d anastrozole, an AI, from 104 patients with ER+ve primary breast cancer [23]. Genes whose expression correlated with expression of *ESR1* levels pre-treatment were identified (Spearman corrected for multiple testing at false discovery rate <1×10^−7^, Table 1 pre-treatment). The mRNA species most highly correlated with *ESR1* were chromosome 6 ORF 97 (*C6ORF97*, Rs = 0.67) (Figure 1a), followed by *C6ORF211*. Other notable inclusions amongst the top 20 most correlated genes included well-established ER-associated genes such as *FOXA1*, *MYB* and *GATA3*, plus *C6ORF96*, also known as *RMND1* (**R**equired for **M**eiotic **N**uclear **D**ivision 1 homolog). The mean pre-treatment expression of the three ORFs was highly correlated with *ESR1* (Rs = 0.70, Figure 1b). After 2 weeks' AI treatment, the top three genes correlating with *ESR1 were C6ORF96, C6ORF97 and C6ORF211* (Rs>0.7 for all, Table 1 two weeks post-treatment). These three ORFs are all located less than 0.5 MB upstream of the *ESR1* start site on the q arm of chromosome 6 (Figure 1e). The expression of other genes located within a 50 MB region surrounding *ESR1* were not correlated with *ESR1* expression (Rs<0.25) (Table S1).

**Table 1:** Genes positively correlated with *ESR1* gene expression ranked according to Spearman correlation.

	GB acc	Gene symbol	Cytoband	Correlation coefficient
	*Pre-treatment*			
**1**	NM_000125	ESR1	6q25.1	1
**2**	NM_025059	C6orf97	6q25.1	0.672
**3**	NM_024573	C6orf211	6q25.1	0.637
**4**	NM_152437	ZNF664	12q24.31	0.608
**5**	NM_019000	FLJ20152	5p15.1	0.562
**6**	NM_015391	ANAPC13	3q22.1	0.552
**7**	NM_018718	TSGA14	7q32	0.547
**8**	NM_017909	C6orf96	6q25.1	0.546
**9**	NM_021627	SENP2	3q27.2	0.545
**10**	NM_012319	SLC39A6	18q12.2	0.544
**11**	NM_004496	FOXA1	14q12-q13	0.537
**12**	NM_005001	NDUFA7	19p13.2	0.534
**13**	NM_207118	GTF2H5	6q25.3	0.532
**14**	NM_004703	RABEP1	17p13.2	0.528
**15**	NM_016058	TPRKB	2p24.3-p24.1	0.528
**16**	NM_005375	MYB	6q22-q23	0.527
**17**	NM_175924	ILDR1	3q13.33	0.526
**18**	NM_173079	RUNDC1	17q21.31	0.526
**19**	NM_032918	RERG	12p12.3	0.523
**20**	NM_002051	GATA3	10p15	0.523
	*2 weeks post-treatment*			
**1**	NM_000125	ESR1	6q25.1	1
**2**	NM_025059	C6orf97	6q25.1	0.741
**3**	NM_017909	C6orf96	6q25.1	0.718
**4**	NM_024573	C6orf211	6q25.1	0.705
**5**	NM_004703	RABEP1	17p13.2	0.688
**6**	NM_006452	PAICS	4q12	0.658
**7**	NM_004496	FOXA1	14q12-q13	0.637
**8**	NM_020784	KIAA1344	14q22.1	0.632
**9**	NM_018199	EXDL2	14q24.1	0.629
**10**	NM_002222	ITPR1	3p26-p25	0.629
**11**	NM_181656	C17orf58	17q24.2	0.625
**12**	NM_002051	GATA3	10p15	0.623
**13**	NM_005080	XBP1	22q12.1|22q12	0.621
**14**	NM_012319	SLC39A6	18q12.2	0.62
**15**	NM_015575	TNRC15	2q37.1	0.619
**16**	NM_173079	RUNDC1	17q21.31	0.615
**17**	NM_015130	TBC1D9	4q31.21	0.608
**18**	NM_138809	LOC134147	5p15.2	0.598
**19**	NM_006405	TM9SF1	14q11.2	0.592
**20**	NM_152416	C8orf38	8q22.1	0.587

All genes shown have parametric p-value and false discovery rates <1e-07.

The correlation was present in all of five published microarray data sets of ER+ve breast cancer in which the C6orfs were included on the array (Table 2). The expression of the three ORFs was lower in ER−ve than ER+ve tumours in the Wang dataset [24] (p = 0.002). No significant correlation was found in the ER−ve subgroup of this dataset. This may be a characteristic of ER−ve tumours or, alternatively, the measurement error associated with low levels of *ESR1* transcript could preclude detection of a significant correlation in microarray data.

**Table 2:** Correlations in other breast cancer datasets.

Study	Number of samples	*C6ORF96*	*C6ORF97*	*C6ORF211*
TransBig [52]	198 breast tumours	0.607	0.776	0.656
Wang – All tumours [24]	286 breast tumours	0.524	0.558	0.769
- ER +ve	209 breast tumours	0.388	0.418	0.608
- ER −ve	65 breast tumours	0.056	0.189	0.087
Loi [35]	354 breast tumours	0.468	0.555	0.588
Huang [53]	23 primary cell lines	0.759	0.759	0.878
Miller [51]	251 breast tumours	0.623	0.547	0.674

Data from five large, publicly available breast cancer datasets performed on Affymetrix U133A arrays which contained probes for *ESR1*, *C6ORF96*, *C6ORF97*, and *C6ORF211* were examined. The mean of all probes for *ESR1* was correlated with each of the three C6ORFs. Correlation co-efficients for each of the genes versus *ESR1* is shown.

### Correlation between *ESR1* and the C6orfs is not explained by amplification

Amplification of the *ESR1* locus has been reported inconsistently [25], [26]. To determine whether the *ESR1*/*C6ORFs* correlation may be the result of underlying genomic co-amplification or deletion events, copy number (CN) status of *ESR1* and the C6orfs was examined using array CGH analysis (resolution 40–60 kb) [27] on DNA from the 44 tumour samples from which adequate further tissue was available. One tumour was shown to be amplified and eight showed gains at *ESR1*, *C6ORF96*, *C6ORF97* and *C6ORF211*, while four showed losses at all four loci. One was measured as having loss of *C6ORF96*, *C6ORF211* and part of *C6ORF97*. While there was some correlation between CN and transcription of the four genes (Figure S1), CN alterations did not explain the correlation between *ESR1* and the C6orfs. In fact, when samples with identified CN changes were removed from the dataset, the correlation between *ESR1* and mean C6orf expression levels strengthened rather than weakened (Rs = 0.83) (Figure 1c), suggesting that transcriptional co-regulation rather than genomic changes is more likely to underlie *ESR1*/*C6ORF* co-expression.

### Change in *ESR1* expression upon aromatase inhibitor treatment is correlated with change in C6orf expression

To assess whether the correlation in *ESR1*/*C6ORF* expression seen in pre-treatment biopsies is reflected in a concordant change in expression of these genes upon treatment, the relationship between the magnitude of change of each of these genes was investigated. Change in expression of *ESR1* induced by aromatase inhibitor treatment over 2 weeks was strongly correlated with change in the C6orfs (Rs = 0.70) (Figure 1d). Given that this short duration of treatment, which has no measurable impact on cellularity or tumour size, is unlikely to facilitate DNA copy number changes throughout the sample this supports the probability that the co-regulation of these genes is at a transcriptional level.

### Expression of *ESR1* and the C6orfs are correlated in MCF7 and BT-474 cells in vitro

**Figure 2:**
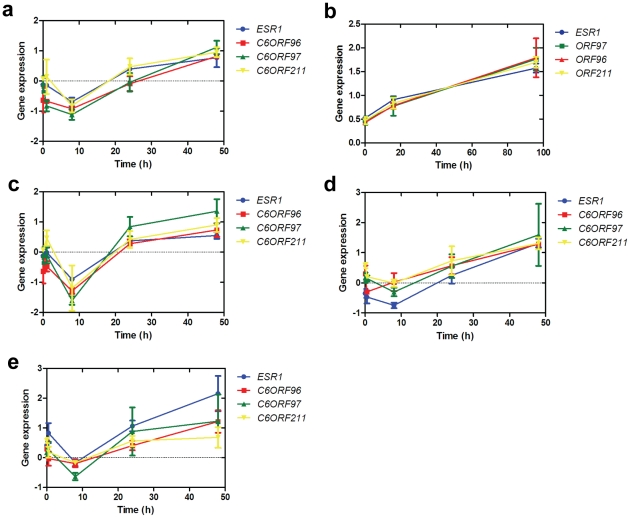
Correlation of C6orf expression *in vitro*. a. Timecourse of expression of *ESR1*, *C6ORF96*, *C6ORF97* and *C6ORF211* in MCF7 cells cultured in the absence of oestradiol. Each gene is normalized to the mean of two housekeeping genes, *TBP* and *FKBP15*. b. Timecourse of expression of *ESR1*, *C6ORF96*, *C6ORF97* and *C6ORF211* in BT-474 cells after addition of lapatinib. c. Timecourse of expression of *ESR1*, *C6ORF96*, *C6ORF97* and *C6ORF211* in MCF7 cells cultured with the addition of ICI 182,780. d. Analysis of expression of nascent *ESR1*, *C6ORF96*, *C6ORF97* and *C6ORF211* in MCF7 cells. e. Analysis of expression of nascent *ESR1*, *C6ORF96*, *C6ORF97* and *C6ORF211* in MCF7 cells treated with ICI. Points represent the mean of three triplicate samples ± SEM.

To determine whether the *ESR1*/*C6ORF* correlations were maintained *in vitro*, transcript levels of ERα and the three C6orfs were measured in oestrogen-deprived MCF7 cells and lapatinib-treated BT-474 cells over a 48- and 96-hour period, respectively. These treatments are both known to have significant effects on the expression of *ESR1*. Lapatinib has been shown to increase ERα in BT-474 cells [28], [29], potentially via loss of Akt and de-repression of FOXO3a. This provides a useful model for manipulation to test the correlation between *ESR1* and the C6orfs *in vitro*. Conversely, absence of oestradiol leads to a short-term reduction in ER expression [30]. Expression of all four genes followed a similar time-course of expression and was highly correlated (Figure 2a and 2b).

ICI 182,780 (ICI) is a steroidal pure anti-oestrogen which causes ERα expression to be suppressed and downregulated [31], [32]. Treatment of MCF7 cells with ICI did not affect ORF expression or their correlation with *ESR1* (Figure 2c). To confirm that the observed correlation was not being influenced by RNA transcribed prior to the addition of ICI, we also measured newly synthesised nascent RNA using PCR amplicons designed to cross an exon/intron boundary [33]. This analysis revealed that nascent transcripts for *ESR1* and the C6orfs remained correlated in both the presence and absence of ICI. The observation that transcription of the genes remains strongly correlated in the presence of ICI suggests that transcriptional regulation by ERα is not the main driver of the *ESR1/C6ORF* co-expression.

### Knockdown of *C6ORF211* by siRNA induces a reduction in proliferation in MCF7cells

**Figure 3:**
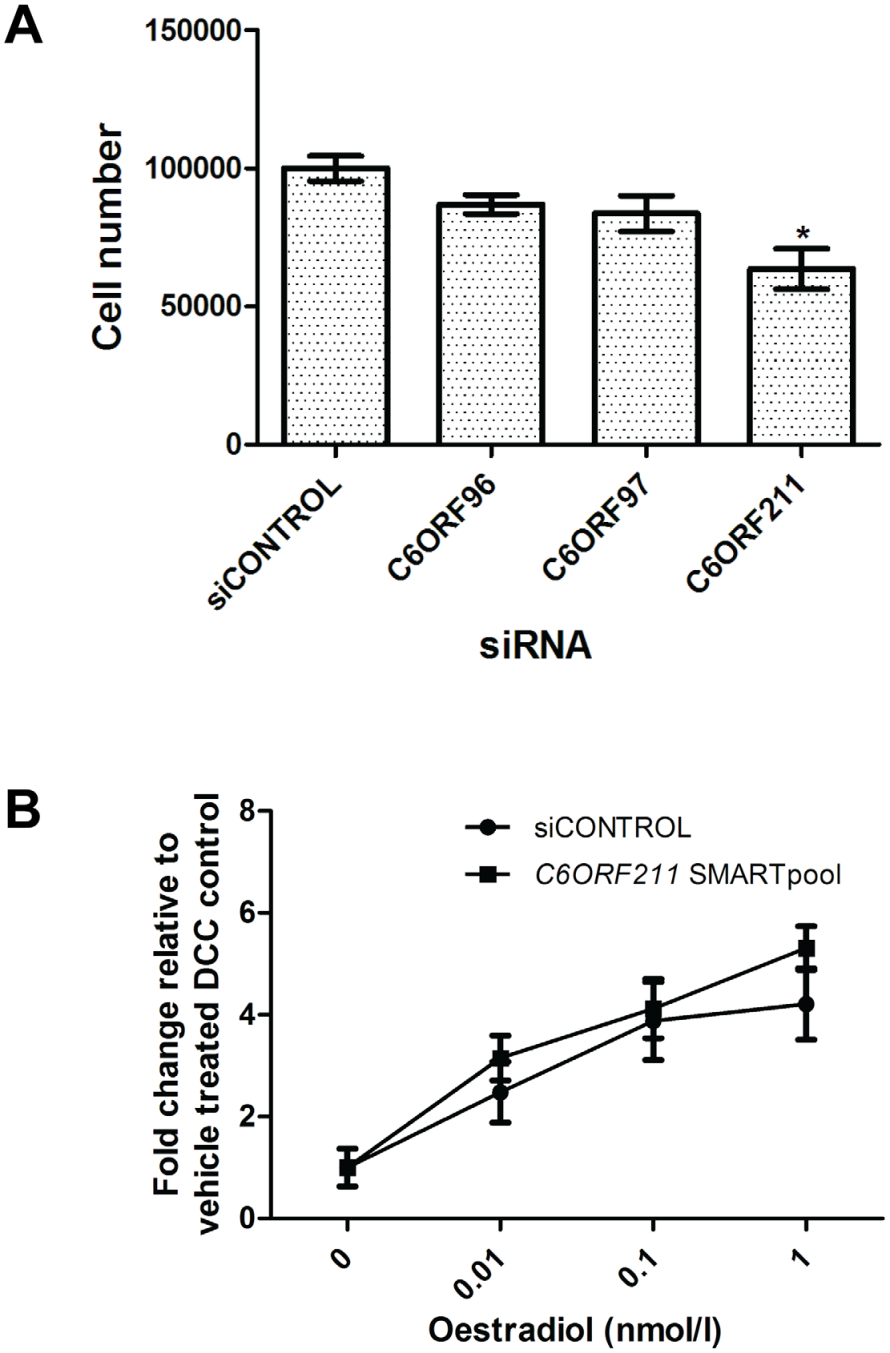
Exploration of the function of the C6orfs in MCF7 cells. a. Wild type-MCF7 cells were stripped of steroid for 48 hours then transfected with either control siRNA, siRNA SMARTpool for *C6ORF96*, *C6ORF97* or *C6ORF211*. b. Stripped MCF7 cells were transfected with *C6ORF211* siRNA SMARTpool and 48 hours post transfection these were treated with increasing concentrations of oestradiol. After 6 days, proliferation in response to siRNA knockdown was established by change in cell number using a Coulter counter. Bars represent the mean ± SEM of four separate repeats of the experiment. Oestradiol-dependent proliferation is shown as fold change relative to cells with no added oestradiol.

The effect of reducing expression of each C6orf on cell proliferation was determined by transfecting siRNA SMARTPOOLs directed against each ORF into MCF7 cells. In cells grown in both E2-containing media and without E2, all three siRNAs reduced transcript levels of their target ORF to <30% of levels in cells transfected with the control non-targeting siRNA pool. Levels of *ESR1*, and the non-targeted ORFs were unaffected by the SMARTpool's (Figure S2) while ESR1-SMARTpool siRNA led to a reduction in levels of all three C6orfs (Figure S3). Immunoblotting with a polyclonal antibody raised against a polypeptide of the predicted product of *C6ORF211* showed an 86% reduction at the protein level (Figure S4). Cells transfected with *C6ORF211* siRNA showed a mean 36% reduction in cell number (p<0.0001) over four separate repeat experiments (Figure 3A). *C6ORF211* knockdown had no effect on oestrogen-dependent proliferation (Figure 3B). Deconvolution of the SMARTPOOL showed that the four constituent siRNAs had a reproducible anti-proliferative effect when compared with scrambled control siRNA (Figure S5). No consistent alteration in proliferation was observed in cells transfected with siRNAs directed against *C6ORF96* or *C6ORF97* (Figure 3A).

### *C6ORF211* correlates with proliferation and clinical outcome in tumours

**Figure 4:**
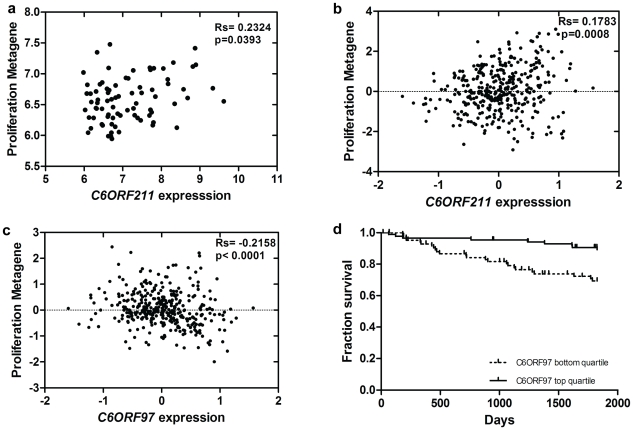
Association between C6orf expression, proliferation, and outcome in tumours. a. Relationship between *C6ORF211* expression and expression of proliferation metagene in 104 breast cancers. b. Relationship between *C6ORF211* expression and expression of proliferation metagene in 354 breast cancers from the Loi dataset. c. Relationship between *C6ORF97* expression and expression of proliferation metagene in the Loi dataset. d. Kaplan–Meier curve representing the fraction relapse-free survival comparing the lowest quartile of *C6ORF97* expression with the highest in the Loi dataset.

To determine whether the association between *C6ORF211* expression and proliferation seen in cultured cells is reflected in tumours, the relationship between *C6ORF211* expression and a metagene composed of known proliferation-associated genes [34] was investigated. In baseline biopsies, levels of *C6ORF211* but not *ESR1* correlated significantly with proliferation (*C6ORF211*, Rs = 0.23, p = 0.04; *ESR1*, Rs = −0.01, p = ns) (Figure 4a), suggesting that *C6ORF211* is more strongly associated with proliferation than *ESR1*. Correlations were also observed with a number of well-known proliferation-associated genes (Table S2). The relationship with proliferation was validated in data from a set of 354 ER+ve tumours [35] (Rs = 0.18, p = 0.0008) (Figure 4b) and the 209 ER+ve tumours from the Wang dataset [24] (Rs = 0.21, p = 0.004). Consistent with the findings in our own data, *ESR1* was not significantly correlated with the proliferation metagene in either of the publicly available datasets (Loi, Rs = −0.03, p = ns; Wang, Rs =  0.02, p = ns). In contrast, *C6ORF97* showed an independent, reproducible negative correlation with proliferation, in our dataset (Rs = −0.19, p = 0.05) and in the Loi (Rs = −0.22, p<0.0001) (Figure 4c) and ER+ve Wang datasets (Rs = −0.24, p = 0.0007).

To determine whether the relationship of the ORFs with proliferation is related to clinical outcome, recurrence free survival (RFS) in tamoxifen-treated patients was investigated for association with *C6ORF97* and *C6ORF211* expression. Despite the fact that in the Loi dataset *ESR1* was not predictive of a significant difference in survival over 5 years [36], the lowest quartile of *C6ORF97* was associated with significantly higher risk of recurrence (HR = 3.1, p = 0.0014) (Figure 4d). A similar trend was observed in untreated ER+ve tumours from the Wang dataset [24], although this was not significant (HR = 1.6, p = 0.16) (Figure S6a). *C6ORF211* was not significantly associated with RFS (Figure S6b and S6c).

## Discussion

Our observation of a previously unreported transcriptional activity hub in the *ESR1*/*C6ORF* region of 6q25.1 has implications for recently identified associations between SNPs in the *ESR1* region and breast cancer risk, as well as broader implications for the biological and clinical importance of ERα in established breast cancer. A number of SNPs, including rs3757318 within intron 7 of *C6ORF97* [7], have been associated with breast cancer risk but the causative variant and mechanism remain undefined [6]–[10]. In an attempt to identify the pathogenic variant, Stacey and colleagues recently reported that GG homozygotes at rs9397435, located immediately downstream of *C6ORF97*, may express higher mean levels of *ESR1* and that the rs9397435 [G] allele conferred significant risk of both hormone receptor positive and hormone receptor negative breast cancer in European and Taiwanese patients [8]. The association of a SNP in this region with ER expression is consistent with findings from our own group which have revealed that the variant genotype of SNP rs2046210 is associated with increased ERα expression as measured by immunohistochemistry [37]. The findings reported in this paper suggest that, due to their high degree of correlation with *ESR1*, levels of *C6ORF97*, *C6ORF96* and *C6ORF211* are also likely to correlate with the rs2046210 and rs9397435 genotype. Consequently, these genes may be involved in the pathogenesis of the variant SNPs and could explain the apparent anomaly noted by Stacey and colleagues in that the SNPs predispose to both hormone receptor positive and negative disease.

To date, analysis of *ESR1* co-expressed genes has focussed on genes which are also downstream targets of the oestradiol-activated transcription factor activity of ERα such as *FOXA1*, *TFF1* and *GATA3*. High throughput technologies have identified numerous classical and novel ERα-dependent targets of oestradiol [11], [17]. This association with the expression of ORFs has, however, not been reported other than by ourselves in abstract form [38].

The transcriptional correlation between *ESR1* and these ORFs is highly statistically significant in our dataset, and in all of the publicly available datasets we examined. In our own patient cohort, we showed that two weeks' treatment with anastrozole induces a concomitant change in *ESR1* and the C6orfs and a yet stronger correlation in their expression. Genomic amplification does not account for the correlations. This suggests that transcriptional co-regulation rather than major genomic rearrangement is likely to underlie their co-expression. To our knowledge, a transcriptional activity hub surrounding a major cancer related gene has not previously been identified.

The observation that the four transcripts remain correlated over a short timecourse in MCF7 and BT474 cells further supports the idea that the co-regulation of these genes is likely to occur at a transcriptional level. Given that ERα can autoregulate its own transcription by binding to an oestrogen responsive element (ERE) in its promoter [17], [39], the possibility that ERα could co-regulate itself and the C6orfs provides an attractive potential explanation for the correlation. We tested this hypothesis by treating MCF7 cells with the ERα antagonist ICI in the absence of E2. Our finding that the nascent transcripts of *ESR1* and the three C6orfs remain correlated in the presence of ICI (Figure 2c) suggests that this co-regulation is not dependent on ERα transcriptional activation.

Regulation of the steady-state level of ERα in breast cancer cells is a complex phenomenon that includes transcriptional and post-transcriptional mechanisms [40]–[42]. *C6ORF96* is transcribed off the opposite DNA strand to *ESR1* (Figure 1e), therefore excluding the possibility that *ESR1* and the ORFs are transcribed as a single polycistronic mRNA. Recent genome-wide mapping experiments have revealed the importance of chromatin organisation for gene expression [18], [43] suggesting that 3-D chromatin arrangement could represent a potential explanation for *C6ORF*/*ESR1* co-expression. However, analysis of the data produced by Fullwood and colleagues [18] shows that *C6ORF96*, *C6ORF97* and *C6ORF211* are not encompassed by an ERα-bound long-range chromatin loop. Nevertheless, it remains possible that a loop driven by an alternative transcription factor could explain the transcriptional activity in this area.

At the nucleotide level, all three ORFs show some homology with *ESR1*, suggesting they may have arisen from gene duplication events [44]. *C6ORF97* encodes a 715 amino acid coiled-coil domain-containing protein that is conserved across 11 species [45] while *C6ORF211* is a member of the UPF0364 protein family of unknown function and is also conserved across multiple species [45]. Confocal analysis revealed that the protein encoded by *C6ORF211* was expressed mainly in the cytoplasm and did not co-localize with ER (Figure S7). In a proteomic screen it has been found to interact with SAP18, a Sin3A-associated cell growth inhibiting protein [46].

This reported interaction with a growth inhibitory protein could explain our observation that knockdown of *C6ORF211* induces suppression of proliferation in cultured cells. This association is mirrored in tumours, where a proliferation metagene correlates significantly with *C6ORF211*. Conversely, *C6ORF97* expression correlates negatively with expression of the proliferation metagene and high *C6ORF97* predicts for improved disease-free survival in a tamoxifen-treated published dataset, independently of *ESR1* (Figure 4d). As high *ESR1* has previously been shown to be associated with improved outcome on endocrine therapy [47], this raises the possibility that, given the observed correlation of *C6ORF97* with *ESR1*, some of this association with outcome could be attributable to *C6ORF97*.

The high degree of correlation between *ESR1* and the C6orfs has significant potential implications for our interpretation of ER levels and therapy of ER+ve breast cancers. As a transducer of mitogenic oestrogen signalling, disruption of ER represents a key target of therapies for ER+ve breast cancer, including tamoxifen and fulvestrant. Our data shows that *C6ORF211* and *C6ORF97* may contribute to the proliferative phenotype of ER+ve tumours, yet these proteins are unlikely to be affected by therapies targeted directly at ERα. Consequently, these proteins may represent potential targets for synergistic therapies in patients with high levels of C6orf expression or targets for breast cancer prevention. In addition, along with further research these relationships could shed light on recent associations between breast cancer risk and SNPs in the region.

## Materials and Methods

### Patient samples

Core-cut tumor biopsies (14-gauge) were obtained from 112 postmenopausal women with stage I to IIIB ER+ early breast cancer before and after two-weeks' anastrozole treatment in a neoadjuvant trial [23]. This study received approval from an institutional review board at each site and was conducted in accordance with the 1964 Declaration of Helsinki [48] and International Conference on Harmonization/Good Clinical Practice guidelines. Written informed consent was obtained from each patient before participation. Tissue was stored in RNAlater at −20°C. Two 4 µm sections from the core were stained with hematoxylin and eosin to confirm the presence of cancerous tissue and the histopathology and six 8 µm sections were retained for microarray CGH analysis (see below). Total RNA was extracted using RNeasy Mini kits (Qiagen, Sussex, UK). RNA quality was checked using an Agilent Bioanalyser (Santa Clara, CA, USA): samples with RNA integrity values of less than 5 were excluded from further analysis. ER status and Ki67 values by immunohistochemistry were already available [23].

### Gene expression analysis and data pre-processing

RNA amplification, labelling and hybridization on HumanWG-6 v2 Expression BeadChips were performed according to the manufacturer's instructions (http://www.illumina.com) at a single Illumina BeadStation facility. Tumor RNA of sufficient quality and quantity was available to generate expression data from 104 pre-treatment biopsies. Data was extracted using BeadStudio software and normalized with variance-stabilizing transformation (VST) and Robust Spline Normalisation method (RSN) in the Lumi package [49]. Probes that were not detected in any samples (detection p value >1%) were discarded from further analysis.

### Data analysis

Multiple correlation analysis was performed in BRB-Array Tools (http://linus.nci.nih.gov/BRB-ArrayTools.html). A statistical significance level for each gene for testing the hypothesis that the Spearman's correlation between expression of *ESR1* and other genes was zero was calculated and p-values were then used in a multivariate permutation test [50] from which false discovery rates were computed. Other statistical analyses were performed in SPSS for Windows (SPSS Inc., Chicago, IL), S-PLUS (TIBCO Software Inc., Palo Alto, CA) and Graphpad Prism (Graphpad Software Inc., La Jolla, CA).

Multivariable analysis was performed in a forward stepwise fashion, the most significant additional variable (satisfying p<0.05) being added at each stage. Cases with missing values for any of the variables in the model were excluded from analysis.

### Analysis of publicly available datasets

For analysis of the breast cancer datasets from public resources the publicly available normalised gene expression data and clinical data were retrieved from Gene Expression Omnibus (http://www.ncbi.nlm.nih.gov/geo/) (‘Wang’ dataset [24], n = 286;GEO, accession number GSE2034) or obtained from the authors (‘Loi’ dataset [35], n = 354 tamoxifen-treated tumours composed of GEO, accession numbers GSE9195, GSE6532 and GSE2990; combined normalised dataset received courtesy of Dr Christos Sotiriou). Correlations between *ESR1* and the C6orfs in the ‘Miller’ [51] (n = 251), ‘TransBig’ (n = 198) [52] and Huang [53] (n = 23 cell lines) were calculated using the correlation analysis tool in Oncomine (http://www.oncomine.org).

Data from the 72 genes comprising the proliferation metagene was retrieved from tumours from the Wang and Loi datasets and proliferation metagene scores were calculated as described previously [54]. Spearman correlation between the proliferation metagene and *ESR1* and the C6orfs was calculated in Graphpad Prism. Survival analysis was carried out in these datasets using the quartiled expression of the C6orfs and the endpoints of recurrence free survival or time to relapse, according to the original publication.

### DNA extraction

Five 8 µm sections from frozen core biopsies were mounted onto Superfrost glass slides, stained with nuclear fast red, and microdissected with a sterile needle under a stereomicroscope to obtain a percentage of tumor cells >75% as described previously [55]. Genomic DNA was extracted as described previously [55]. The concentration of the DNA was measured with Picogreen according to the manufacturer's instructions (Invitrogen).

### Array CGH analysis

The 32K bacterial artificial chromosome (BAC) re-array collection (CHORI) tiling path aCGH platform used for this study was constructed in the Breakthrough Breast Cancer Research Centre [55]. DNA labelling, array hybridisations, image acquisition and filtering were performed as described in Natrajan et al. [56]. Data were smoothed using the circular binary segmentation (cbs) algorithm [27]. A categorical analysis was applied to the BACs after classifying them as representing gain, loss or no-change according to their smoothed Log2 ratio values as defined [56].

### Cell culture

MCF7 cells were routinely maintained in phenol red free RPMI1640 (Invitrogen, Paisley, UK) supplemented with 10% foetal bovine serum and oestradiol (1 nM). Cells were passaged weekly and medium replenished every 48–72 hours. In the case of BT474, cell monolayers were cultured in phenol red containing medium supplemented with 10% foetal bovine serum. Cell lines were shown to be free of mycoplasma by routine testing.

### Real-time quantitative PCR

Total RNA from treated MCF7 and BT-474 cells was extracted using the RNeasy Mini Kit (Qiagen) according to the manufacturer's instructions. All RNA quantification was performed using the Agilent 2100 Bioanalyzer with RNA Nano LabChip Kits (Agilent Technologies, Wokingham, Berkshire, UK). RNA was reverse transcribed using SuperScript III (Invitrogen), and random primers. Twenty nanograms of resulting cDNA of each sample was analyzed in triplicates by qRT-PCR using the ABI Perkin-Elmer Prism 7900HT Sequence detection system (Applied Biosystems). Taqman gene expression assays (Applied Biosystems) were used to quantitate processed transcripts of *ESR1* (Hs01046818_m1), *C6ORF96* (Hs00215537_m1), *C6ORF97* (Hs01563344_m1), *C6ORF211* (Hs00226188_m1), which were normalized to two housekeeping genes, *FKBP15* (Hs00391480_m1) and *TBP* (Hs00427620_m1). These housekeepers were selected from a previously published list of appropriate reference genes for breast cancer [57]. Custom assays using primers designed to span intron-exon boundaries were used to measure nascent RNA (Table S3). Gene expression was quantified using a standard curve generated from serial dilutions of reference cDNA from a pooled breast cancer cell line RNA.

### Immunoblots

Cell monolayers were washed with cold PBS twice and collected by scraping. Cell pellets were lysed in extraction buffer, resolved by SDS-PAGE and transferred to nitrocellulose membranes as described previously [30]. Membranes were blocked and probed with a polyclonal antibody directed against the predicted peptide (amino acids 368–382) of C6orf211 (Eurogentec, Southampton, UK) and anti β-actin (Sigma-Aldrich, Poole, UK) using the methods described previously [58]. Quantification of immunoblots was performed using the NIH ImageJ software, and immunoblots were normalized to actin.

### Immunofluorescence and confocal studies

Cells were grown on glass coverslips in standard growth medium. Cells were fixed and incubated in the presence of primary antibodies as described previously [58]. Coverslips were washed with PBS and cells were incubated in the presence of appropriate Alexa Fluor 555 (red) or Alexa Fluor 488 (green)-labeled secondary antibodies (Molecular Probes, Invitrogen, Paisley, UK) diluted 1∶1000 for 1 hr. Cells were washed in PBS and nuclei (DNA) were counterstained with 4,6-diamidino-2-phenylindole (DAPI; Invitrogen) diluted 1∶10000. Coverslips were mounted onto glass slides using Vectashield mounting medium (Vector Laboratories, Peterborough, UK). Images were collected sequentially in three channels on a Zeiss LSM710 (Carl Zeiss Ltd, Welwyn Garden City, UK) laser scanning confocal microscope at the same magnification (×63 oil immersion objective).

### Cell proliferation assays

Cell lines were depleted of steroids for 3 days by culturing in DCC-medium [59], seeded into 12-well plates at a density of 1×10^4^ cells/well for MCF7 and 4×10^4^ cells per well for BT474, monolayers were allowed to acclimatize for 24 h before treatment with drug combinations indicated for 6 d with daily changes. Cell number was determined using a Z1 Coulter Counter (Beckman Coulter). Results were confirmed in a minimum of three independent experiments, and each experiment was performed in triplicate.

### Effect of oestradiol and ICI182780 on ORF RNA expression

Wt-MCF7 cells were stripped of steroid for 3 days as described above. Cells were subsequently seeded into 12 well plates at a density of 1×10^5^ cells/well. After 24 hours monolayers were treated with vehicle (0.01% v/v ethanol), oestradiol (1 nM) or ICI182780 (10 nM) for the time intervals indicated. RNA was extracted using RNeasy Mini kit (Qiagen) and subjected to qRT-PCR as described.

### SiRNA knockdown of ORFs

Wt-MCF7 cells were stripped of steroid for 24 hours in DCC-medium. Stripped cells were subsequently seeded into 12 well plates at a density of 2×10^4^ cells/well for proliferation assays or 1×10^5^ cells/well for RNA expression analysis. After 24 hours monolayers were transfected with 100 nM of either siRNA against *C6ORF96*, *C6ORF97*, *C6ORF211* or control siRNA using DharmaFECT 1 reagent (Dharmacon, Thermo Fisher Scientific, UK). Medium was then replenished the following day and cells were allowed to acclimatise for a further 24 hours. After 24 hours samples were taken for RNA expression analysis. For analysis of oestrogen-dependent proliferation, the monolayers were treated with increasing concentrations of oestradiol (0.01, 0.1 or 1 nM) 48 hours post transfection. The remaining plates were treated daily with the treatments indicated for 6 days before carrying out cell counts as described above.

## Supporting Information

Figure S1 Correlation between *ESR1* and the mean of *C6ORF96*, *C6ORF97*, and *C6ORF211* showing tumours with measured copy number variations shown in colour.
(0.18 MB DOC)

Figure S2 Validation of C6ORF gene silencing by siRNA. MCF7 cells were grown in either media containing stripped serum or stripped serum plus 1 nM oestradiol and transfected with siRNA. After 48 h, RNA was extracted from cells and complementary DNA synthesized using standard methods. Using Assay-on-Demand primer/probe sets (Applied Biosystems, UK), we performed real-time quantitative PCR. Gene expression was calculated relative to expression of *TBP* and *FKBP15* and adjusted relative to expression in cells transfected with a non-targeting siRNA (siControl). Error bars represent the standard error of the mean (SEM). MCF7 cells were transfected with siRNA against *C6ORF96*, *C6ORF97*, *C6ORF211* or control siRNA in A. DCC or B. 1 nM oestradiol.
(0.78 MB DOC)

Figure S3 Validation of *C6ORF211* gene silencing in deconvolution of siRNA SMARTPOOL. MCF7 cells were grown in media containing stripped serum and transfected with individual siRNAs. After 48 h, RNA was extracted from cells and complementary DNA synthesized using standard methods. Using Assay-on-Demand primer/probe sets (Applied Biosystems, UK), we performed real-time quantitative PCR. Gene expression was calculated relative to expression of *TBP* and *FKBP15* and adjusted relative to expression in cells transfected with a non-targeting siRNA (siRNA Control). Error bars represent the standard error of the mean (SEM).
(0.84 MB DOC)

Figure S4 Validation of C6ORF protein knockdown by siRNA. MCF7 cells were transfected with siRNA against *C6ORF97*, *C6ORF211* or control siRNA. 72 h after siRNA transfection, cell lysates were generated and immunoblotted using a. a polyclonal antibody generated against C6orf211 and b. anti-β-actin as a loading control.
(0.06 MB DOC)

Figure S5 Validation of proliferation changes induced by individual siRNAs. WT-MCF7 cells were stripped of steroid for 24 hours in DCC-medium. Stripped cells were seeded into 12 well plates at a density of 20,000 cells/well for proliferation assays or 100,000 cells/well for RNA expression analysis. After 24 hours monolayers were transfected with 100 nM of single siRNAs against *C6ORF211* or control siRNA (SMARTPool). Medium was replenished the following day and cells were allowed to acclimatise for a further 24 hours. Monolayers were subsequently treated with fresh DCC medium. The remaining plates were treated with DCC medium for 6 days. Proliferation in response to individual siRNA knockdown were established by change in cell number using a coulter counter (Beckman Scientific UK). Data presented is expressed as absolute cell number or fold change over siControl (SMARTpool). All data is from triplicate wells, each well read twice.
(0.27 MB DOC)

Figure S6 a. Kaplan-Meier curve comparing proportion relapse-free survival in the lowest quartile of *C6ORF97* expression versus the highest in 142 untreated ER+ve tumours from the Wang dataset. b. Kaplan-Meier curve comparing the proportion relapse-free survival in the lowest quartile of *C6ORF211* expression versus the highest in 345 tamoxifen-treated ER+ve tumours from the Loi dataset. c. Kaplan-Meier curve comparing the proportion relapse-free survival in the lowest quartile of *C6ORF211* expression versus the highest in 142 untreated ER+ve tumours from the Wang dataset.
(0.69 MB DOC)

Figure S7 Confocal analysis of C6orf211 localisation. To determine the subcellular localization of C6orf211 protein, confocal analysis was carried out using a polyclonal antibody directed against the predicted peptide (amino acids 368–381). MCF-7 cells were plated onto coverslips and stained. a. Nuclei were visualized using DAPI and stained with antibodies against C6ORF211 (b) and oestrogen receptor (c). An overlay of all three images is shown in (d).
(0.07 MB DOC)

Table S1 Correlation of expression of genes in the region of amplification surrounding *ESR1* as defined by Reis-Filho et al. (2008) [26] with expression of *ESR1* in baseline biopsies from 104 patients with ER+ve breast cancer.
(0.06 MB DOC)

Table S2 Correlation expression of the C6ORFs and *ESR1* with expression of well-known proliferation genes. Correlations significant at p<0.05 are indicated with an asterisk.
(0.04 MB DOC)

Table S3 Custom assays designed to measure nascent RNA.
(0.04 MB DOC)

## References

[1] Parkin DM, Bray F, Ferlay J, Pisani P (2005). Global cancer statistics, 2002.. CA Cancer J Clin.

[2] Dowsett M, Dunbier AK (2008). Emerging biomarkers and new understanding of traditional markers in personalized therapy for breast cancer.. Clin Cancer Res.

[3] Perou CM, Sorlie T, Eisen MB, van de Rijn M, Jeffrey SS (2000). Molecular portraits of human breast tumours.. Nature.

[4] Hammes SR, Levin ER (2007). Extranuclear steroid receptors: nature and actions.. Endocr Rev.

[5] Sorlie T, Tibshirani R, Parker J, Hastie T, Marron JS (2003). Repeated observation of breast tumor subtypes in independent gene expression data sets.. Proc Natl Acad Sci U S A.

[6] Zheng W, Long J, Gao YT, Li C, Zheng Y (2009). Genome-wide association study identifies a new breast cancer susceptibility locus at 6q25.1.. Nat Genet.

[7] Turnbull C, Ahmed S, Morrison J, Pernet D, Renwick A (2010). Genome-wide association study identifies five new breast cancer susceptibility loci.. Nat Genet.

[8] Stacey SN, Sulem P, Zanon C, Gudjonsson SA, Thorleifsson G (2010). Ancestry-Shift Refinement Mapping of the C6orf97-ESR1 Breast Cancer Susceptibility Locus.. PLoS Genet.

[9] Fletcher O, Johnson N, Orr N, Hosking FJ, Gibson LJ (2011). Novel Breast Cancer Susceptibility Locus at 9q31.2: Results of a Genome-Wide Association Study.. J Natl Cancer Inst.

[10] Cai Q, Wen W, Qu S, Li G, Egan KM (2011). Replication and Functional Genomic Analyses of the Breast Cancer Susceptibility Locus at 6q25.1 Generalize Its Importance in Women of Chinese, Japanese, and European Ancestry.. Cancer Res.

[11] Frasor J, Danes JM, Komm B, Chang KC, Lyttle CR (2003). Profiling of estrogen up- and down-regulated gene expression in human breast cancer cells: insights into gene networks and pathways underlying estrogenic control of proliferation and cell phenotype.. Endocrinology.

[12] Oh DS, Troester MA, Usary J, Hu Z, He X (2006). Estrogen-regulated genes predict survival in hormone receptor-positive breast cancers.. J Clin Oncol.

[13] Yu J, Yu J, Cordero KE, Johnson MD, Ghosh D (2008). A transcriptional fingerprint of estrogen in human breast cancer predicts patient survival.. Neoplasia.

[14] Miller WR, Larionov AA, Renshaw L, Anderson TJ, White S (2007). Changes in breast cancer transcriptional profiles after treatment with the aromatase inhibitor, letrozole.. Pharmacogenet Genomics.

[15] Mackay A, Urruticoechea A, Dixon JM, Dexter T, Fenwick K (2007). Molecular response to aromatase inhibitor treatment in primary breast cancer.. Breast Cancer Res.

[16] Mello-Grand M, Singh V, Ghimenti C, Scatolini M, Regolo L (2010). Gene expression profiling and prediction of response to hormonal neoadjuvant treatment with anastrozole in surgically resectable breast cancer.. Breast Cancer Res Treat.

[17] Carroll JS, Meyer CA, Song J, Li W, Geistlinger TR (2006). Genome-wide analysis of estrogen receptor binding sites.. Nat Genet.

[18] Fullwood MJ, Liu MH, Pan YF, Liu J, Xu H (2009). An oestrogen-receptor-alpha-bound human chromatin interactome.. Nature.

[19] Rae JM, Johnson MD, Scheys JO, Cordero KE, Larios JM (2005). GREB 1 is a critical regulator of hormone dependent breast cancer growth.. Breast Cancer Res Treat.

[20] Shang Y, Hu X, DiRenzo J, Lazar MA, Brown M (2000). Cofactor dynamics and sufficiency in estrogen receptor-regulated transcription.. Cell.

[21] Musgrove EA, Sutherland RL (2009). Biological determinants of endocrine resistance in breast cancer.. Nat Rev Cancer.

[22] Ali S, Coombes RC (2002). Endocrine-responsive breast cancer and strategies for combating resistance.. Nat Rev Cancer.

[23] Smith IE, Walsh G, Skene A, Llombart A, Mayordomo JI (2007). A phase II placebo-controlled trial of neoadjuvant anastrozole alone or with gefitinib in early breast cancer.. J Clin Oncol.

[24] Wang Y, Klijn JG, Zhang Y, Sieuwerts AM, Look MP (2005). Gene-expression profiles to predict distant metastasis of lymph-node-negative primary breast cancer.. Lancet.

[25] Holst F, Stahl PR, Ruiz C, Hellwinkel O, Jehan Z (2007). Estrogen receptor alpha (ESR1) gene amplification is frequent in breast cancer.. Nat Genet.

[26] Reis-Filho JS, Drury S, Lambros MB, Marchio C, Johnson N (2008). ESR1 gene amplification in breast cancer: a common phenomenon?. Nat Genet.

[27] Mackay A, Tamber N, Fenwick K, Iravani M, Grigoriadis A (2009). A high-resolution integrated analysis of genetic and expression profiles of breast cancer cell lines.. Breast Cancer Res Treat.

[28] Xia W, Bacus S, Hegde P, Husain I, Strum J (2006). A model of acquired autoresistance to a potent ErbB2 tyrosine kinase inhibitor and a therapeutic strategy to prevent its onset in breast cancer.. Proc Natl Acad Sci U S A.

[29] Leary AF, Martin L, Thornhill A, Dowsett M, Johnston S (2010). Combining or Sequencing Targeted Therapies in ER+/HER2 Amplified Breast Cancer (BC): In Vitro and In Vivo Studies of Letrozole and Lapatinib in an ER+/HER2+ Aromatase-Transfected BC Model.. Cancer Res.

[30] Martin LA, Farmer I, Johnston SR, Ali S, Marshall C (2003). Enhanced estrogen receptor (ER) alpha, ERBB2, and MAPK signal transduction pathways operate during the adaptation of MCF-7 cells to long term estrogen deprivation.. J Biol Chem.

[31] Wakeling AE, Bowler J (1992). ICI 182,780, a new antioestrogen with clinical potential.. J Steroid Biochem Mol Biol.

[32] McClelland RA, Gee JM, Francis AB, Robertson JF, Blamey RW (1996). Short-term effects of pure anti-oestrogen ICI 182780 treatment on oestrogen receptor, epidermal growth factor receptor and transforming growth factor-alpha protein expression in human breast cancer.. Eur J Cancer.

[33] Li GJ, Zhao Q, Zheng W (2005). Alteration at translational but not transcriptional level of transferrin receptor expression following manganese exposure at the blood-CSF barrier in vitro.. Toxicol Appl Pharmacol.

[34] Ghazoui Z, Buffa FM, Dunbier AK, Anderson H, Dexter T (2011). Close and stable relationship between proliferation and a hypoxia metagene in aromatase inhibitor treated ER-positive breast cancer.. Clin Cancer Res.

[35] Loi S, Haibe-Kains B, Desmedt C, Wirapati P, Lallemand F (2008). Predicting prognosis using molecular profiling in estrogen receptor-positive breast cancer treated with tamoxifen.. BMC Genomics.

[36] Loi S, Haibe-Kains B, Desmedt C, Lallemand F, Tutt AM (2007). Definition of clinically distinct molecular subtypes in estrogen receptor-positive breast carcinomas through genomic grade.. J Clin Oncol.

[37] Drury S, Johnson N, Hills M, Salter J, Dunbier A (2010). A breast cancer-associated SNP adjacent to ESR1 correlates with oestrogen receptor-alpha (ER alpha) level in invasive breast tumours.. Cancer Res.

[38] Dunbier AK, Anderson H, Ghazoui Z, Pancholi S, Sidhu K (2010). ESR1 is co-expressed with closely adjacent novel genes in estrogen receptor positive breast cancer..

[39] Lazennec G, Huignard H, Valotaire Y, Kern L (1995). Characterization of the transcription start point of the trout estrogen receptor-encoding gene: evidence for alternative splicing in the 5′ untranslated region.. Gene.

[40] Martin MB, Saceda M, Garcia-Morales P, Gottardis MM (1994). Regulation of estrogen receptor expression.. Breast Cancer Res Treat.

[41] Martin MB, Angeloni SV, Garcia-Morales P, Sholler PF, Castro-Galache MD (2004). Regulation of estrogen receptor-alpha expression in MCF-7 cells by taxol.. J Endocrinol.

[42] Angeloni SV, Martin MB, Garcia-Morales P, Castro-Galache MD, Ferragut JA (2004). Regulation of estrogen receptor-alpha expression by the tumor suppressor gene p53 in MCF-7 cells.. J Endocrinol.

[43] Lieberman-Aiden E, van Berkum NL, Williams L, Imakaev M, Ragoczy T (2009). Comprehensive mapping of long-range interactions reveals folding principles of the human genome.. Science.

[44] http://www.genecards.org/

[45] http://www.ncbi.nlm.nih.gov/homologene

[46] Ewing RM, Chu P, Elisma F, Li H, Taylor P (2007). Large-scale mapping of human protein-protein interactions by mass spectrometry.. Mol Syst Biol.

[47] Early Breast Cancer Trialists' Collaborative Group (1998). Tamoxifen for early breast cancer: an overview of the randomised trials.. Lancet.

[48] The World Medical Association: Declaration of Helsinki. http://www.wma.net/e/policy/b3htm

[49] Du P, Kibbe WA, Lin SM (2008). lumi: a pipeline for processing Illumina microarray.. Bioinformatics.

[50] Korn EL, Troendle JF, McShane LM, Simon R (2004). Controlling the number of false discoveries: application to high-dimensional genomic data.. Journal of Statistical Planning and Inference.

[51] Miller LD, Smeds J, George J, Vega VB, Vergara L (2005). An expression signature for p53 status in human breast cancer predicts mutation status, transcriptional effects, and patient survival.. Proc Natl Acad Sci U S A.

[52] Desmedt C, Piette F, Loi S, Wang Y, Lallemand F (2007). Strong time dependence of the 76-gene prognostic signature for node-negative breast cancer patients in the TRANSBIG multicenter independent validation series.. Clin Cancer Res.

[53] Huang F, Reeves K, Han X, Fairchild C, Platero S (2007). Identification of candidate molecular markers predicting sensitivity in solid tumors to dasatinib: rationale for patient selection.. Cancer Res.

[54] Ghazoui Z, Buffa FM, Dunbier AK, Anderson H, Dexter T (2009). Aromatase Inhibitors Reduce the Expression of a Hypoxia Metagene in Oestrogen Receptor Positive Breast Cancer in Postmenopausal Women.. Cancer Res.

[55] Marchio C, Iravani M, Natrajan R, Lambros MB, Savage K (2008). Genomic and immunophenotypical characterization of pure micropapillary carcinomas of the breast.. J Pathol.

[56] Natrajan R, Weigelt B, Mackay A, Geyer FC, Grigoriadis A (2010). An integrative genomic and transcriptomic analysis reveals molecular pathways and networks regulated by copy number aberrations in basal-like, HER2 and luminal cancers.. Breast Cancer Res Treat.

[57] Drury S, Anderson H, Dowsett M (2009). Selection of REFERENCE genes for normalization of qRT-PCR data derived from FFPE breast tumors.. Diagn Mol Pathol.

[58] Pancholi S, Lykkesfeldt AE, Hilmi C, Banerjee S, Leary A (2008). ERBB2 influences the subcellular localization of the estrogen receptor in tamoxifen-resistant MCF-7 cells leading to the activation of AKT and RPS6KA2.. Endocr Relat Cancer.

[59] Darbre P, Yates J, Curtis S, King RJ (1983). Effect of estradiol on human breast cancer cells in culture.. Cancer Res.

