# Supplementary material for: ESR1 Is Co-Expressed with Closely Adjacent Uncharacterised Genes Spanning a Breast Cancer Susceptibility Locus at 6q25.1
Source: PLoS Genet. 2011 Apr 28;7(4):e1001382. doi: 10.1371/journal.pgen.1001382 (PMC3084198; doi:10.1371/journal.pgen.1001382)
Supplement: Figure S1 — Correlation between ESR1 and the mean of C6ORF96, C6ORF97, and C6ORF211 showing tumours with measured copy number variations shown in colour. (0.18 MB DOC) [file pgen.1001382.s001.doc]

**Figure S1.** Correlation between *ESR1* and the mean of *C6ORF96*, *C6ORF97* and *C6ORF211* showing tumours with measured copy number variations shown in colour.


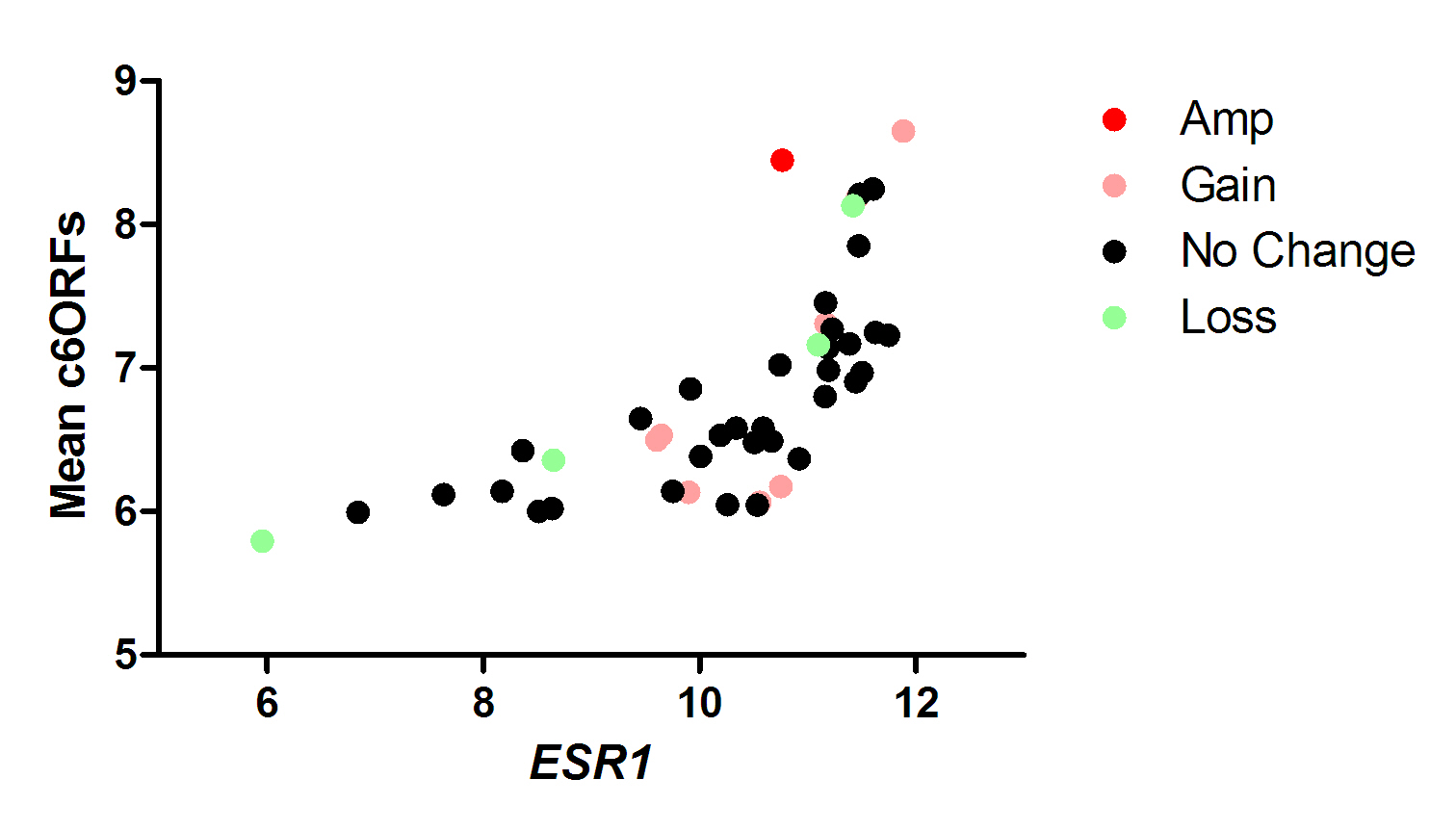


**Rs=0.8219**

**p < 0.0001**

Deletion
