# Supplementary material for: ESR1 Is Co-Expressed with Closely Adjacent Uncharacterised Genes Spanning a Breast Cancer Susceptibility Locus at 6q25.1
Source: PLoS Genet. 2011 Apr 28;7(4):e1001382. doi: 10.1371/journal.pgen.1001382 (PMC3084198; doi:10.1371/journal.pgen.1001382)
Supplement: Figure S2 — Validation of C6ORF gene silencing by siRNA. MCF7 cells were grown in either media containing stripped serum or stripped serum plus 1 nM oestradiol and transfected with siRNA. After 48 h, RNA was extracted from cells and complementary DNA synthesized using standard methods. Using Assay-on-Demand primer/probe sets (Applied Biosystems, UK), we performed real-time quantitative PCR. Gene expression was calculated relative to expression of TBP and FKBP15 and adjusted relative to expression in cells transfected with a non-targeting siRNA (siControl). Error bars represent the standard error of the mean (SEM). MCF7 cells were transfected with siRNA against C6ORF96, C6ORF97, C6ORF211 or control siRNA in A. DCC or B. 1 nM oestradiol. (0.78 MB DOC) [file pgen.1001382.s002.doc]

**Figure S2.** Validation of C6orf gene silencing by siRNA. MCF7 cells were grown in either media containing stripped serum or stripped serum plus 1 nM oestradiol and transfected with siRNA. After 48 h, RNA was extracted from cells and complementary DNA synthesized using standard methods. Using Assay-on-Demand primer/probe sets (Applied Biosystems, UK), we performed real-time quantitative PCR. Gene expression was calculated relative to expression of *TBP* and *FKBP15* and adjusted relative to expression in cells transfected with a non-targeting siRNA (siControl). Error bars represent the standard error of the mean (SEM). MCF7 cells were transfected with siRNA against *C6ORF96*, *C6ORF97*, *C6ORF211* or control siRNA in **A.** DCC or **B.** 1 nM oestradiol.


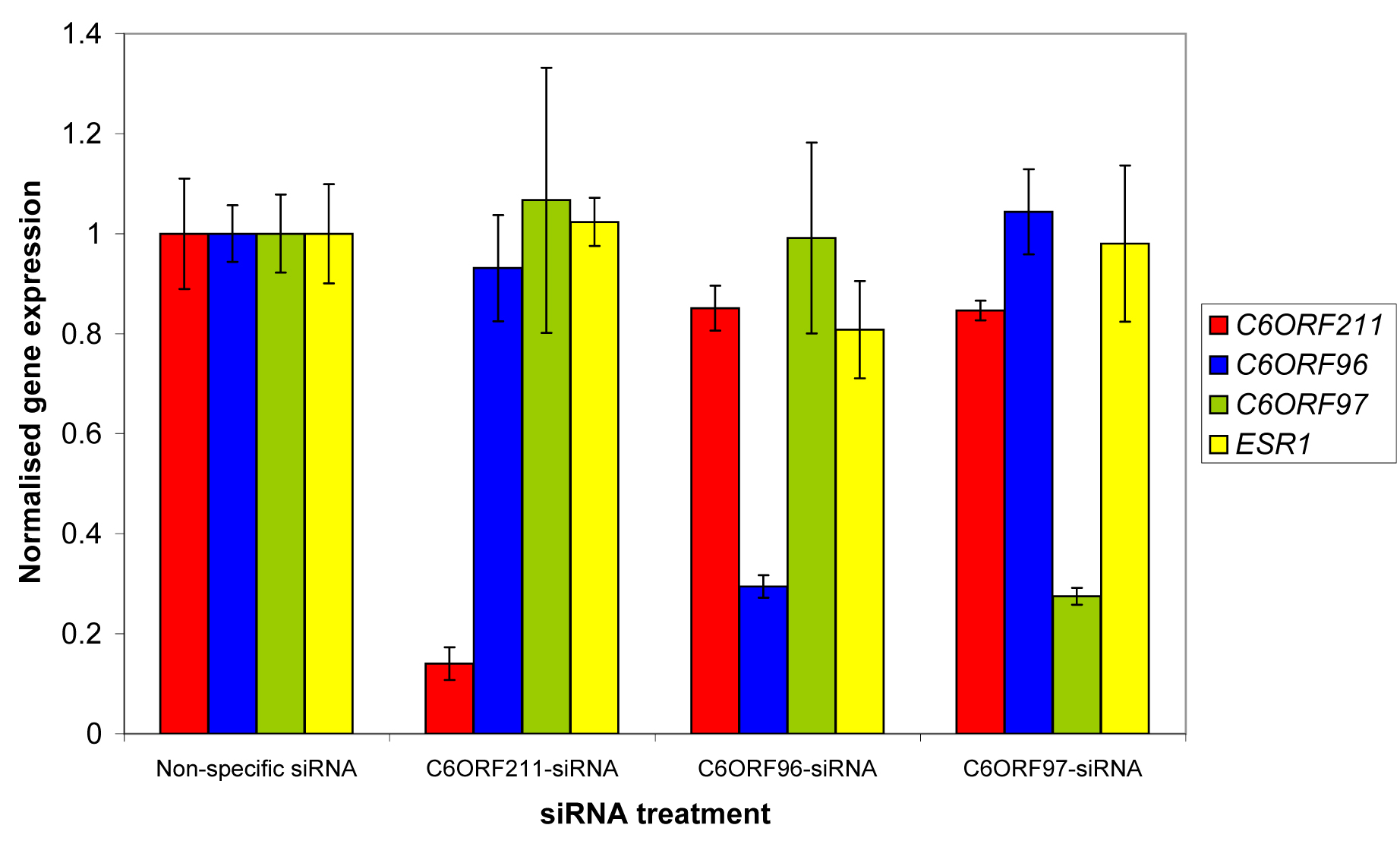


**A.**


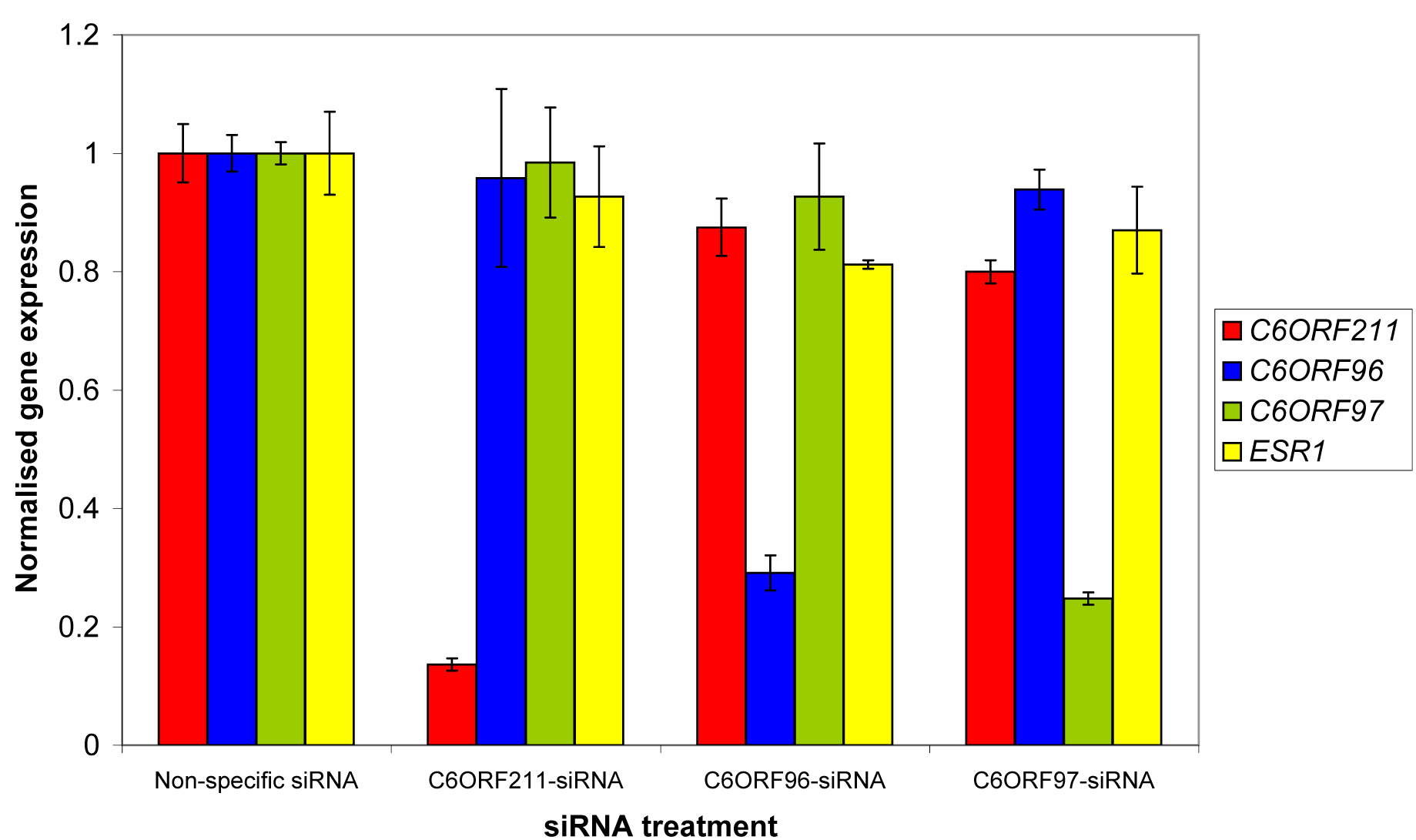


**B.**
