# Supplementary material for: ESR1 Is Co-Expressed with Closely Adjacent Uncharacterised Genes Spanning a Breast Cancer Susceptibility Locus at 6q25.1
Source: PLoS Genet. 2011 Apr 28;7(4):e1001382. doi: 10.1371/journal.pgen.1001382 (PMC3084198; doi:10.1371/journal.pgen.1001382)

**Figure S3.** Validation of *C6ORF211* gene silencing in deconvolution of siRNA SMARTPOOL. MCF7 cells were grown in media containing stripped serum and transfected with individual siRNAs. After 48 h, RNA was extracted from cells and complementary DNA synthesized using standard methods. Using Assay-on-Demand primer/probe sets (Applied Biosystems, UK), we performed real-time quantitative PCR. Gene expression was calculated relative to expression of *TBP* and *FKBP15* and adjusted relative to expression in cells transfected with a non-targeting siRNA (siRNA Control). Error bars represent the standard error of the mean (SEM).


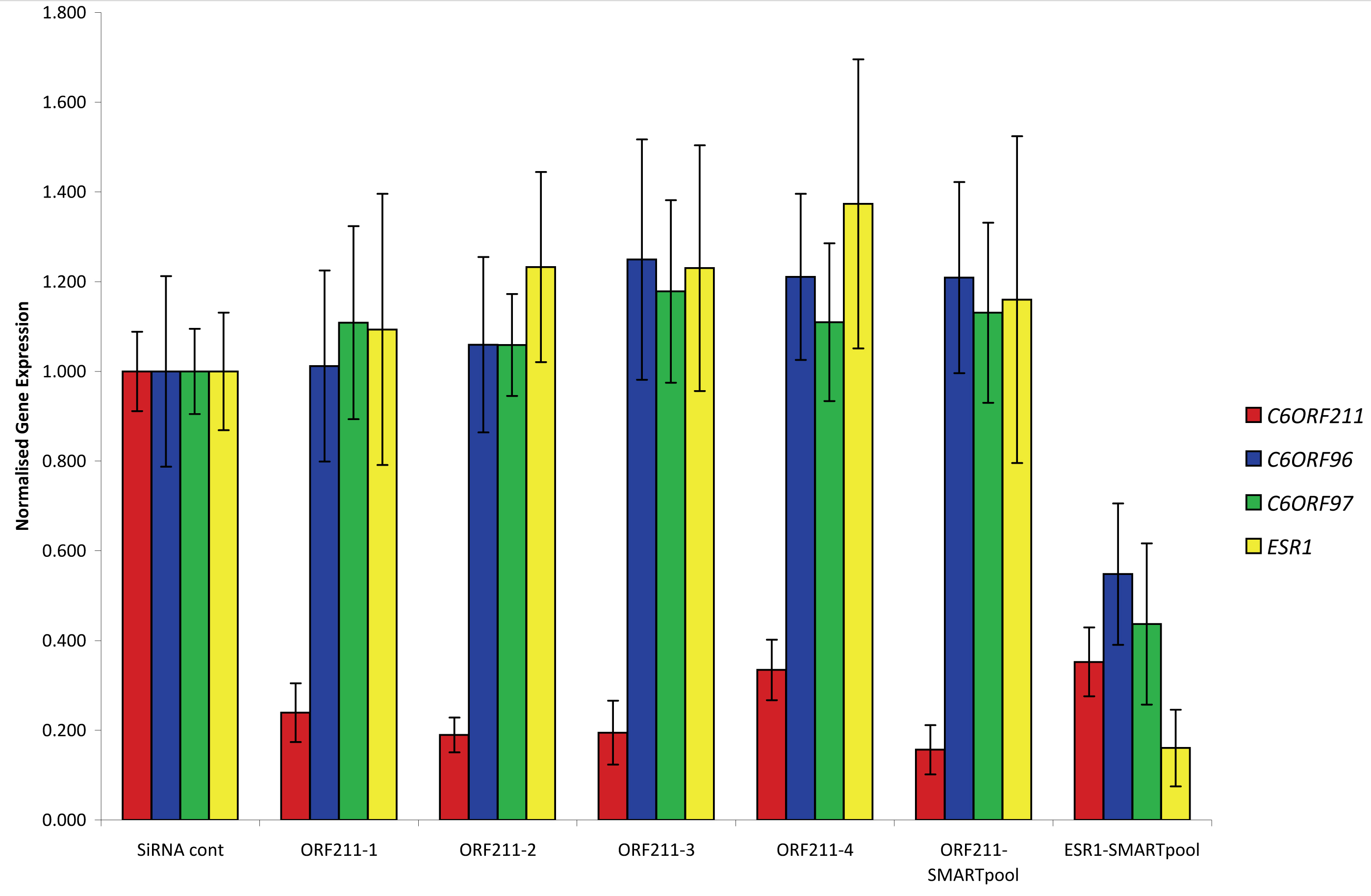

Supplement: Figure S3 — Validation of C6ORF211 gene silencing in deconvolution of siRNA SMARTPOOL. MCF7 cells were grown in media containing stripped serum and transfected with individual siRNAs. After 48 h, RNA was extracted from cells and complementary DNA synthesized using standard methods. Using Assay-on-Demand primer/probe sets (Applied Biosystems, UK), we performed real-time quantitative PCR. Gene expression was calculated relative to expression of TBP and FKBP15 and adjusted relative to expression in cells transfected with a non-targeting siRNA (siRNA Control). Error bars represent the standard error of the mean (SEM). (0.84 MB DOC) [file pgen.1001382.s003.doc]
