# Supplementary material for: ESR1 Is Co-Expressed with Closely Adjacent Uncharacterised Genes Spanning a Breast Cancer Susceptibility Locus at 6q25.1
Source: PLoS Genet. 2011 Apr 28;7(4):e1001382. doi: 10.1371/journal.pgen.1001382 (PMC3084198; doi:10.1371/journal.pgen.1001382)

**Figure S4.** Validation of C6orf protein knockdown by siRNA. MCF7 cells were transfected with siRNA against *C6ORF97*, *C6ORF211* or control siRNA. 72 h after siRNA transfection, cell lysates were generated and immunoblotted using **a.** a polyclonal antibody generated against C6orf211 and **b.** anti-β-actin as a loading control.


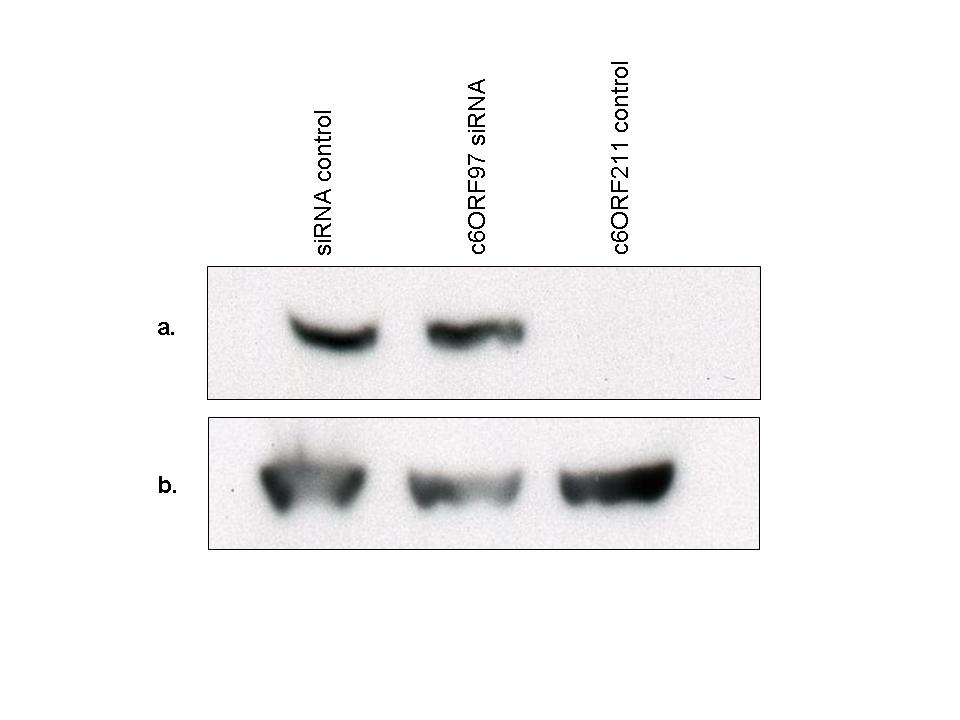

Supplement: Figure S4 — Validation of C6ORF protein knockdown by siRNA. MCF7 cells were transfected with siRNA against C6ORF97, C6ORF211 or control siRNA. 72 h after siRNA transfection, cell lysates were generated and immunoblotted using a. a polyclonal antibody generated against C6orf211 and b. anti-β-actin as a loading control. (0.06 MB DOC) [file pgen.1001382.s004.doc]
