# Supplementary material for: ESR1 Is Co-Expressed with Closely Adjacent Uncharacterised Genes Spanning a Breast Cancer Susceptibility Locus at 6q25.1
Source: PLoS Genet. 2011 Apr 28;7(4):e1001382. doi: 10.1371/journal.pgen.1001382 (PMC3084198; doi:10.1371/journal.pgen.1001382)

**Figure S5.** Validation of proliferation changes induced by individual siRNAs. WT-MCF7 cells were stripped of steroid for 24 hours in DCC-medium. Stripped cells were seeded into 12 well plates at a density of 20,000 cells/well for proliferation assays or 100,000 cells/well for RNA expression analysis. After 24 hours monolayers were transfected with 100nM of single siRNAs against *ORF211* or control siRNA (SMARTPool). Medium was replenished the following day and cells were allowed to acclimatise for a further 24 hours. Monolayers were subsequently treated with fresh DCC medium. The remaining plates were treated with DCC medium for 6 days. Proliferation in response to individual siRNA knockdown were established by change in cell number using a coulter counter (Beckman Scientific UK). Data presented is expressed as absolute cell number or fold change over siControl (SMARTpool). All data is from triplicate wells, each well read twice.

**A**


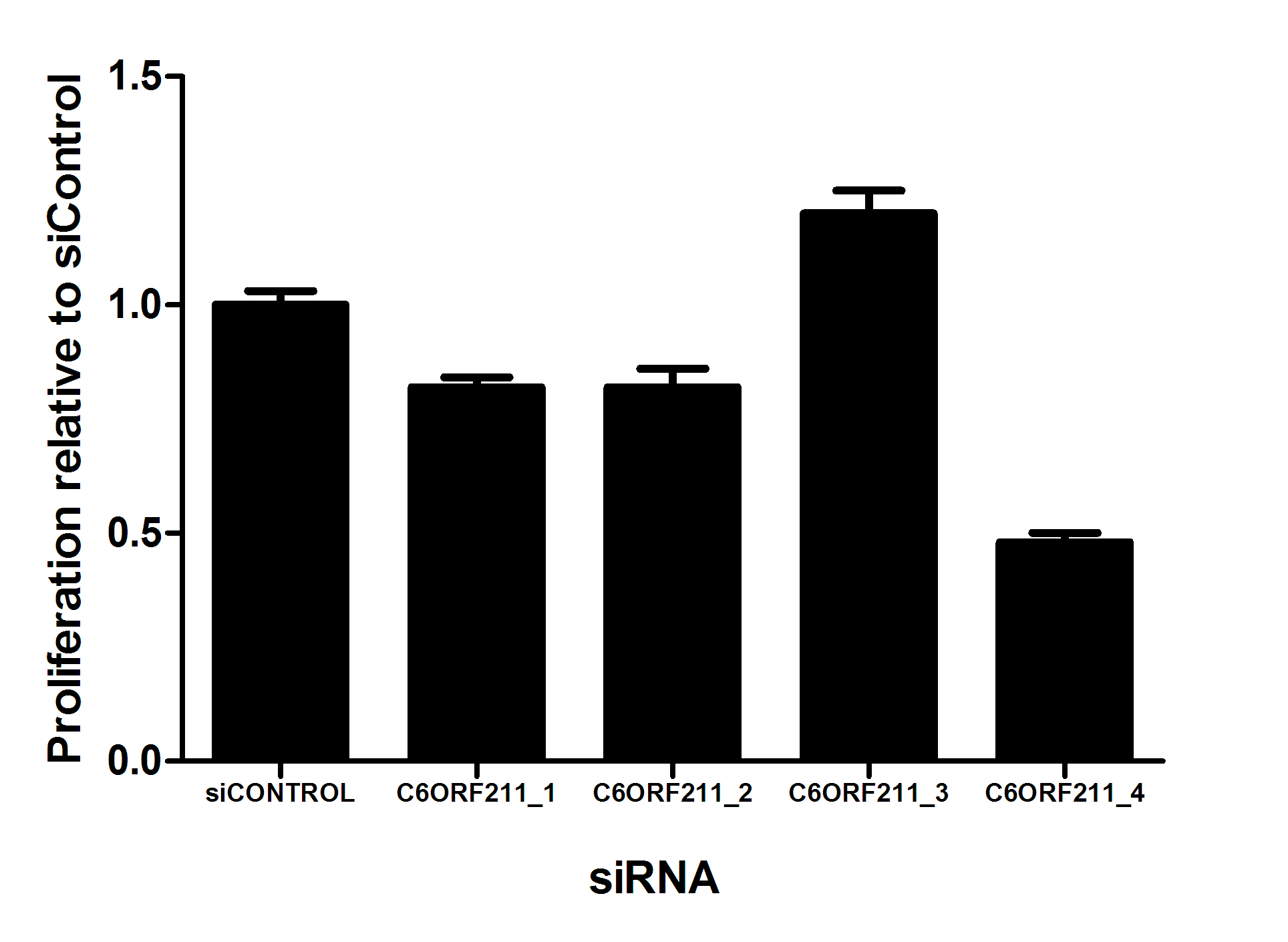

Supplement: Figure S5 — Validation of proliferation changes induced by individual siRNAs. WT-MCF7 cells were stripped of steroid for 24 hours in DCC-medium. Stripped cells were seeded into 12 well plates at a density of 20,000 cells/well for proliferation assays or 100,000 cells/well for RNA expression analysis. After 24 hours monolayers were transfected with 100 nM of single siRNAs against C6ORF211 or control siRNA (SMARTPool). Medium was replenished the following day and cells were allowed to acclimatise for a further 24 hours. Monolayers were subsequently treated with fresh DCC medium. The remaining plates were treated with DCC medium for 6 days. Proliferation in response to individual siRNA knockdown were established by change in cell number using a coulter counter (Beckman Scientific UK). Data presented is expressed as absolute cell number or fold change over siControl (SMARTpool). All data is from triplicate wells, each well read twice. (0.27 MB DOC) [file pgen.1001382.s005.doc]
