# Supplementary material for: ESR1 Is Co-Expressed with Closely Adjacent Uncharacterised Genes Spanning a Breast Cancer Susceptibility Locus at 6q25.1
Source: PLoS Genet. 2011 Apr 28;7(4):e1001382. doi: 10.1371/journal.pgen.1001382 (PMC3084198; doi:10.1371/journal.pgen.1001382)
Supplement: Figure S6 — a. Kaplan-Meier curve comparing proportion relapse-free survival in the lowest quartile of C6ORF97 expression versus the highest in 142 untreated ER+ve tumours from the Wang dataset. b. Kaplan-Meier curve comparing the proportion relapse-free survival in the lowest quartile of C6ORF211 expression versus the highest in 345 tamoxifen-treated ER+ve tumours from the Loi dataset. c. Kaplan-Meier curve comparing the proportion relapse-free survival in the lowest quartile of C6ORF211 expression versus the highest in 142 untreated ER+ve tumours from the Wang dataset. (0.69 MB DOC) [file pgen.1001382.s006.doc]

**Figure S6**. **a.** Kaplan–Meier curve comparing proportion relapse-free survival in the lowest quartile of *C6ORF97* expression versus the highest in 142 untreated ER+ve tumours from the Wang dataset. **b.** Kaplan–Meier curve comparing the proportion relapse-free survival in the lowest quartile of *C6ORF211* expression versus the highest in 345 tamoxifen-treated ER+ve tumours from the Loi dataset. **c.** Kaplan–Meier curve comparing the proportion relapse-free survival in the lowest quartile of *C6ORF211* expression versus the highest in 142 untreated ER+ve tumours from the Wang dataset.


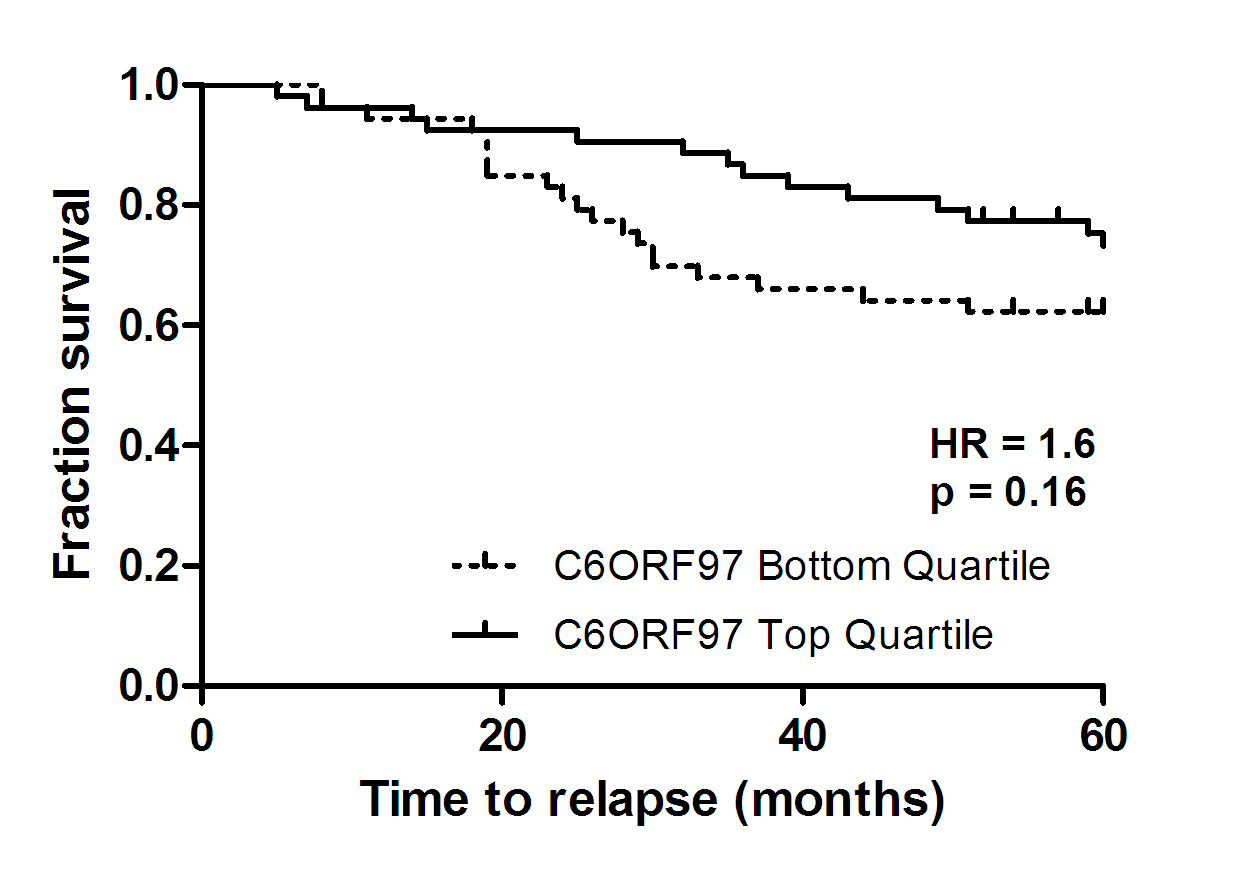


**a.**


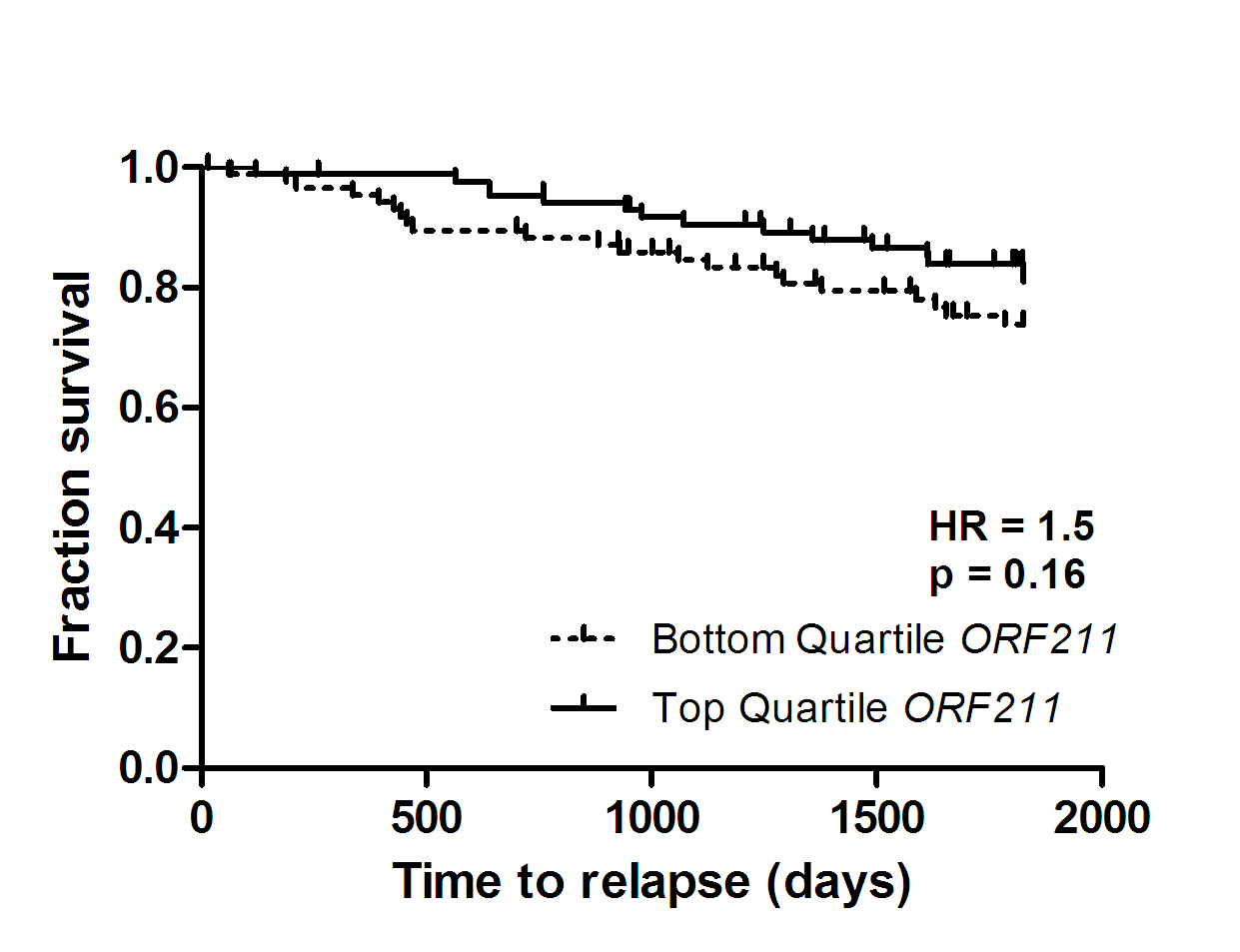


**b.**


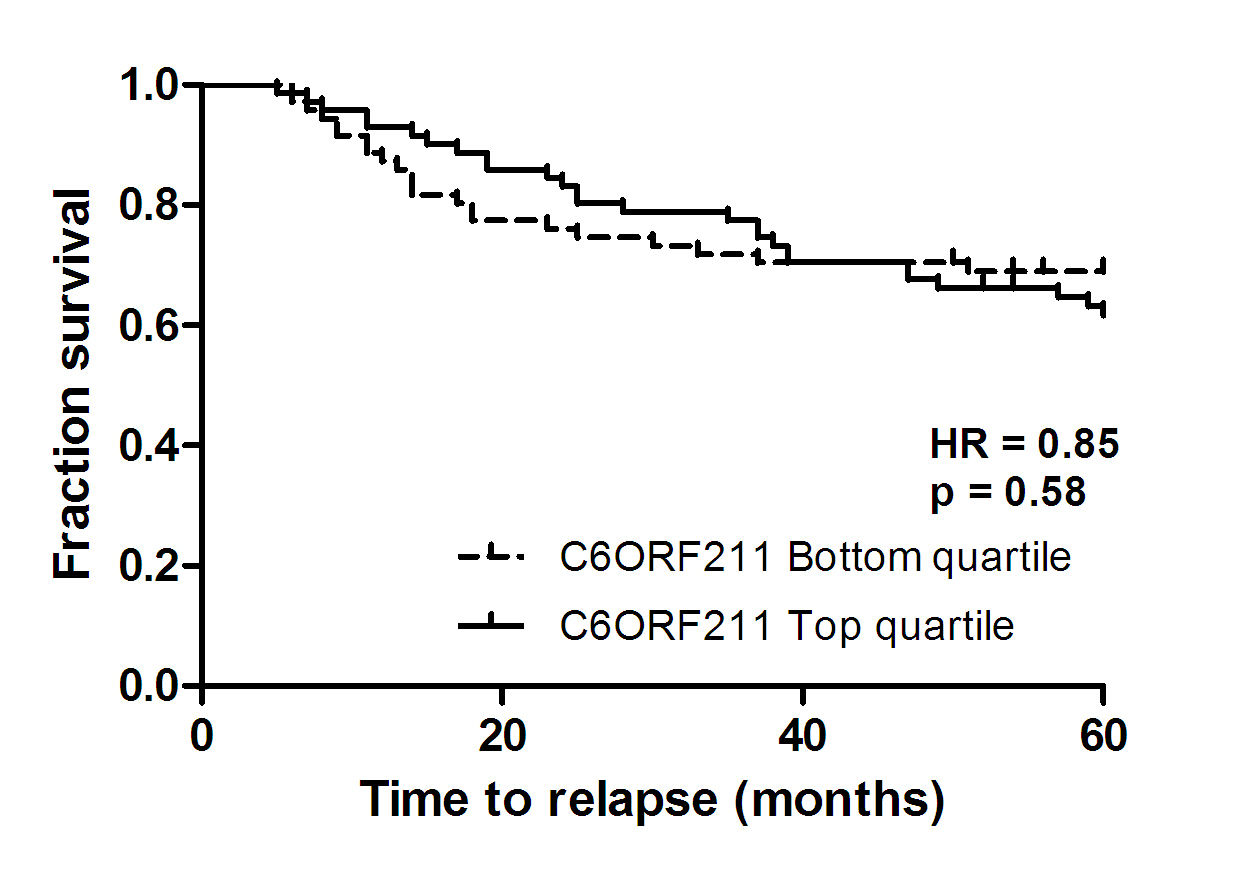


**c.**

**b.**
