# Supplementary material for: ESR1 Is Co-Expressed with Closely Adjacent Uncharacterised Genes Spanning a Breast Cancer Susceptibility Locus at 6q25.1
Source: PLoS Genet. 2011 Apr 28;7(4):e1001382. doi: 10.1371/journal.pgen.1001382 (PMC3084198; doi:10.1371/journal.pgen.1001382)
Supplement: Figure S7 — Confocal analysis of C6orf211 localisation. To determine the subcellular localization of C6orf211 protein, confocal analysis was carried out using a polyclonal antibody directed against the predicted peptide (amino acids 368–381). MCF-7 cells were plated onto coverslips and stained. a. Nuclei were visualized using DAPI and stained with antibodies against C6ORF211 (b) and oestrogen receptor (c). An overlay of all three images is shown in (d). (0.07 MB DOC) [file pgen.1001382.s007.doc]

**Figure S7. Confocal analysis of C6orf211 localisation.** To determine the subcellular localization of C6orf211 protein, confocal analysis was carried out using a polyclonal antibody directed against the predicted peptide (amino acids 368-381). MCF-7 cells were plated onto coverslips and stained. **a.** Nuclei were visualized using DAPI and stained with antibodies against C6orf211 (**b**) and oestrogen receptor (**c**). An overlay of all three images is shown in (**d**).

**
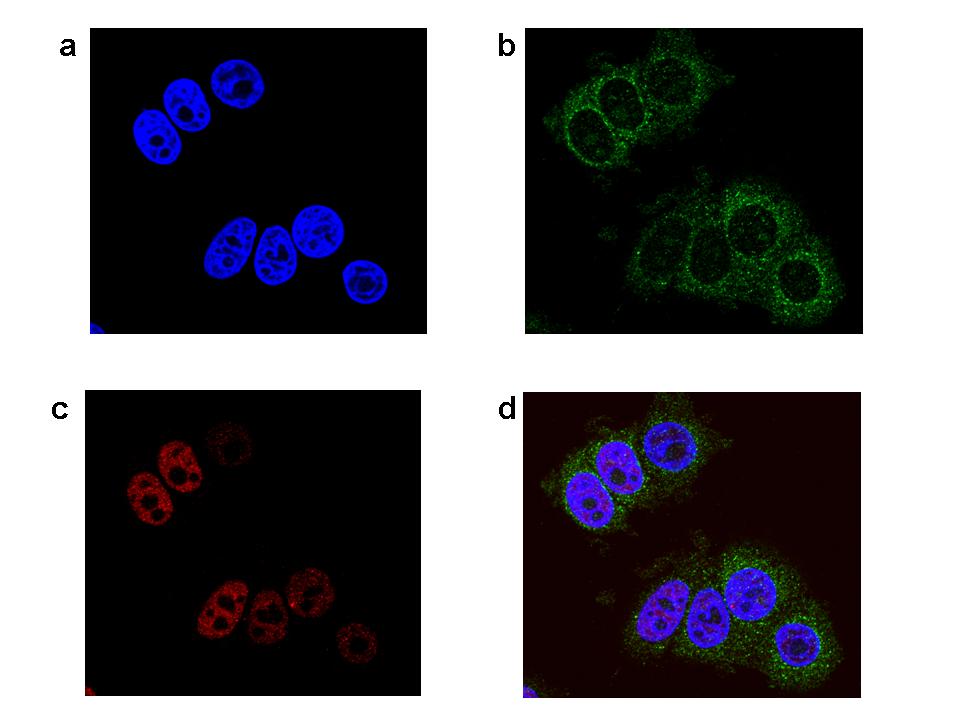
**
