# Supplementary material for: ESR1 Is Co-Expressed with Closely Adjacent Uncharacterised Genes Spanning a Breast Cancer Susceptibility Locus at 6q25.1
Source: PLoS Genet. 2011 Apr 28;7(4):e1001382. doi: 10.1371/journal.pgen.1001382 (PMC3084198; doi:10.1371/journal.pgen.1001382)
Supplement: Table S1 — Correlation of expression of genes in the region of amplification surrounding ESR1 as defined by Reis-Filho et al. (2008) [26] with expression of ESR1 in baseline biopsies from 104 patients with ER+ve breast cancer. (0.06 MB DOC) [file pgen.1001382.s008.doc]

**Supplementary Table 1:** Correlation of expression of genes in the region of amplification surrounding *ESR1* as defined by Reis-Filho *et al*., 2008 with expression of ESR1 in baseline biopsies from 104 patients with ER+ve breast cancer.

| **UGRepAcc** | **Parameter** | **Genomic location** | **Spearman r** | **P value (two-tailed)** |
| --- | --- | --- | --- | --- |
| NM_000125 | ESR1 | chr6:152243483-152423952 |  |  |
| NM_015293 | SYNE1 | chr6:152698107-152744190 | -0.00581 | 0.9564 |
| NM_025059 | C6ORF97 | chr6:151856868-151984020 | 0.6953 | p<0.0001 |
| NM_024573 | C6ORF211 | chr6:151,815,115-151,832,925 | 0.6489 | p<0.0001 |
| NM_017909 | C6ORF96 | chr6:151,799,065-151,808,653 | 0.5781 | p<0.0001 |
| NM_020861 | ZBTB2 | chr6:151726943-151754370 | 0.1829 | 0.0826 |
| NM_005100 | AKAP12 | chr6:151,688,359-151,721,385 | -0.2204 | 0.0358 |
| NM_015440 | MTHFD1L | chr6:151238072-151464716 | -0.2419 | 0.0209 |
| NM_001029884 | PLEKHG1 | chr6:151083917-151195034 | -0.07308 | 0.4912 |
| NM_203395 | IYD | chr6:150731721-150767457 | -0.1215 | 0.2511 |
| NM_030949 | PPP1R14C | chr6:150,505,881-150,613,221 | -0.2328 | 0.0264 |
| NM_024518 | ULBP3 | chr6:150427436-150431976 | -0.06671 | 0.5298 |
| NM_130900 | RAET1L | chr6:150382959-150388361 | 0.002293 | 0.9828 |
| NM_025218 | ULBP1 | chr6:150326836-150336539 | -0.02064 | 0.846 |
| NM_025217 | ULBP2 | chr6:150304829-150312061 | -0.1396 | 0.1868 |
| NM_139165 | RAET1E | chr6:150251294-150253790 | 0.04181 | 0.694 |
| NM_001001788 | RAET1G | chr6:150279707-150285907 | -0.02865 | 0.7875 |
| NM_032832 | LRP11 | chr6:150181587-150227173 | 0.2492 | 0.0172 |
| NM_005389 | PCMT1 | chr6:150112524-150174249 | 0.1344 | 0.2041 |
| NM_024647 | NUP43 | chr6:150087150-150109381 | 0.1251 | 0.2375 |
| NM_007044 | KATNA1 | chr6:149957865-150001421 | 0.0308 | 0.7719 |
| NM_138785 | C6ORF72 | chr6:149929221-149953760 | 0.1688 | 0.1098 |
| NM_139126 | PPIL4 | chr6:149867324-149908864 | -0.1318 | 0.2131 |
| NM_145342 | MAP3K7IP2 | chr6:149732738-149773954 | -0.1218 | 0.2501 |
| NM_001002255 | SUMO4 | chr6:149763188-149763875 | -0.05483 | 0.6057 |
