# Supplementary material for: ESR1 Is Co-Expressed with Closely Adjacent Uncharacterised Genes Spanning a Breast Cancer Susceptibility Locus at 6q25.1
Source: PLoS Genet. 2011 Apr 28;7(4):e1001382. doi: 10.1371/journal.pgen.1001382 (PMC3084198; doi:10.1371/journal.pgen.1001382)
Supplement: Table S2 — Correlation expression of the C6ORFs and ESR1 with expression of well-known proliferation genes. Correlations significant at p<0.05 are indicated with an asterisk. (0.04 MB DOC) [file pgen.1001382.s009.doc]

**Supplementary Table 2:** Correlation expression of the C6ORFs and ESR1 with expression of well-known proliferation genes. Correlations significant at p<0.05 are indicated with an asterisk.

|  | *C6ORF96* | *C6ORF97* | *C6ORF211* | Average *C6ORF* | *ESR1* |
| --- | --- | --- | --- | --- | --- |
| *MKI67* | 0.24* | 0.00 | 0.23* | 0.21* | 0.06 |
| *AURKA* | 0.19 | -0.05 | 0.09 | 0.10 | 0.05 |
| *CCNB1* | 0.22* | -0.13 | 0.11 | 0.12 | 0.05 |
| *CCND1* | 0.28* | 0.17 | 0.26* | 0.30* | 0.32* |
| *CCND2* | -0.29* | -0.16 | -0.06 | -0.17 | -0.25* |
| *CCNE1* | 0.08 | -0.14 | 0.11 | 0.06 | -0.04 |
| *CCNE2* | 0.13 | 0.06 | 0.08 | 0.12 | 0.04 |
