# Supplementary material for: ESR1 Is Co-Expressed with Closely Adjacent Uncharacterised Genes Spanning a Breast Cancer Susceptibility Locus at 6q25.1
Source: PLoS Genet. 2011 Apr 28;7(4):e1001382. doi: 10.1371/journal.pgen.1001382 (PMC3084198; doi:10.1371/journal.pgen.1001382)
Supplement: Table S3 — Custom assays designed to measure nascent RNA. (0.04 MB DOC) [file pgen.1001382.s010.doc]

**Supplementary Table 3:** Custom assays designed to measure nascent RNA

| Gene | Location | Forward primer (5’→3’) | Reverse primer | Probe |
| --- | --- | --- | --- | --- |
| *C6ORF96* | Intron 4, Exon 4 junction | CCTTGACATAGCTTTTTTGTTCGTT | TTCCACACCCATCACCAAAAT | TTAAATCACAGATGCAGCAAA |
| *C6ORF211* | Exon 3, Intron 3 junction | ACCGTGGTTGTTGGTAGAATGTT | CACACGTACCTCTGGATAATTGCT | ATGTATCGAAGAATTCATG |
| *C6ORF97* | Exon 9, Intron 9 junction | GCCGACACCAATGAACTGAA | AACCAGCAAAAAGCAACAACAA | AAGTGCTTGGCTTCAT |
| *ESR1* | Intron 8, Exon 9 junction | GCCGACACCAATGAACTGAA | AACCAGCAAAAAGCAACAACAA | AAGTGCTTGGCTTCAT |
